# Enzyme association for environmental biotransformation reactions through contrastive learning of reaction center-specific fingerprints

**DOI:** 10.1093/bioinformatics/btag142

**Published:** 2026-03-24

**Authors:** Kunyang Zhang, Thierry D Marti, Silke I Probst, Serina L Robinson, Kathrin Fenner

**Affiliations:** Department of Environmental Chemistry, Eawag, Dübendorf 8600, Switzerland; Department of Chemistry, University of Zürich, Zürich 8057, Switzerland; Department of Environmental Microbiology, Eawag, Dübendorf 8600, Switzerland; Department of Environmental Systems Science, ETH Zürich, Zürich 8057, Switzerland; Department of Environmental Microbiology, Eawag, Dübendorf 8600, Switzerland; Department of Environmental Microbiology, Eawag, Dübendorf 8600, Switzerland; Department of Environmental Systems Science, ETH Zürich, Zürich 8057, Switzerland; Department of Environmental Chemistry, Eawag, Dübendorf 8600, Switzerland; Department of Chemistry, University of Zürich, Zürich 8057, Switzerland

## Abstract

**Motivation:**

Microbial biotransformation plays a central role in the environmental degradation of chemical contaminants, driven by the catalytic activities of diverse enzymes. However, linking specific enzymes to contaminant removal and predicting associated transformation products (TPs) under real-world conditions remain a major challenge. In this study, we present a self-supervised, contrastive fine-tuning strategy for reaction fingerprint learning, designed to improve the chemical relevance of BERT-based reaction embeddings for environmental biotransformation reactions. Specifically, we fine-tuned a BERT encoder such that the cosine similarity between its reaction fingerprints aligns with the Tanimoto similarity of traditional structure-based fingerprints.

**Results:**

The resulting compact, 256-dimensional fingerprints, termed crxnfp, showed an improved ability to cluster reactions according to transformation type and focus attention on chemically meaningful reaction centers. Our crxnfp fingerprints were further validated in reaction classification tasks across multiple datasets, achieving superior or comparable performance relative to existing methods. Importantly, they enabled a similarity-based association of biotransformation rules and reactions from enviPath with enzyme annotations from the Rhea and UniProt databases, offering a scalable approach to enrich environmental biotransformation datasets with enzymatic information. Additionally, crxnfp was employed to identify specific enzyme classes involved in contaminant biotransformation, which were subsequently validated through experiments conducted in this study, achieving 91.3% accuracy at the third-level enzyme classification. The crxnfp fingerprints offer a promising solution to advance the understanding of contaminant biotransformation and guide the development of enzyme-informed strategies for contaminant management across diverse environmental contexts.

**Availability and implementation:**

Code is available at https://github.com/zhangky12/crxnfp and https://github.com/zhangky12/crxnfp_knn.

## 1 Introduction

Microorganisms play a crucial role in transforming chemical contaminants into less bioactive forms or achieving complete mineralization (Fenner *et al.* 2021). Among various possible degradation processes, biotransformation is considered the most important in terms of mass balance, compared to abiotic processes like photolysis or chemical oxidation (Fenner *et al.* 2013). Biotransformation activity is largely driven by broad-spectrum enzymes capable of catalyzing diverse chemical reactions (Fenner *et al.* 2013). With advances in techniques such as metagenomic sequencing (Tringe *et al.* 2005), comprehensive profiling of genes that encode enzymes in environmental samples has become widely accessible. However, identifying causal links between contaminant removal and specific biotransformation agents, including microbes and their associated enzymatic pathways, remains a major challenge.

Efforts to associate enzymes with reactions profit from the development of curated resources such as the Rhea database (Bansal *et al.* 2022) and the UniProt database (UniProt Consortium 2019). Rhea is a comprehensive resource of expert-curated biochemical reactions, with detailed reaction information and support for multiple reaction formats. It serves as an important platform for connecting chemical transformations with enzyme functions (Bansal *et al.* 2022). Rhea reactions are cross-linked to entries in the UniProt database, which is a widely used repository for protein sequence and functional information, including enzyme classification, gene ontology annotations, and known biochemical activities (UniProt Consortium 2019). The mapping between Rhea and UniProt enables researchers to connect specific reaction entries with their catalyzing enzymes, supporting functional annotation and pathway analysis. Extrapolating from such known enzyme-reaction associations to novel reactions is an active area of research, and several tools have emerged to predict enzyme annotations based on reaction input (Yamanishi *et al.* 2009, Dalkiran *et al.* 2018, Li *et al.* 2018, Yu *et al.* 2023, Yang *et al.* 2024). In the environmental context, the enviPath database offers a complementary perspective focused on microbial biotransformation pathways for environmental contaminants (Wicker *et al.* 2016). EnviPath includes a rich set of biotransformation rules and reactions, some of which are linked to enzymes via the enviLink extension (Schmid *et al.* 2021). However, many of the newer or predicted reactions in enviPath still lack enzyme information. Predicting enzymes for these orphan reactions and transformation rules in databases such as enviPath can help bridge this gap.

**Figure 1:**
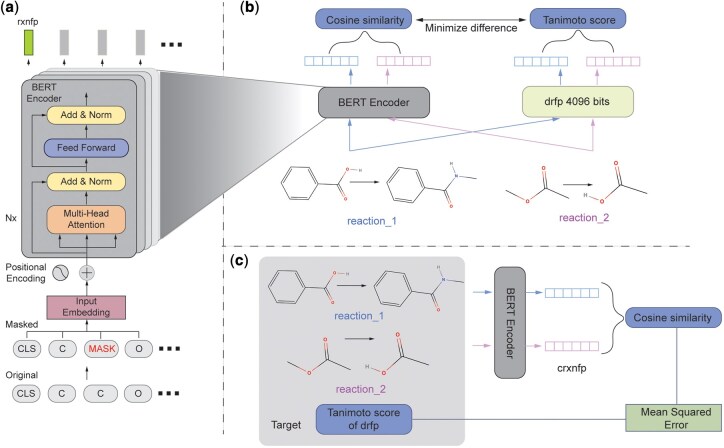
Model architecture. (a) The original BERT model trained using a masked language modeling objective to generate rxnfp fingerprints, where the embedding of the [CLS] token is used as the fingerprint representation. (b) The fine-tuning objective is to align the cosine similarity of reaction fingerprints produced by the BERT encoder with the Tanimoto similarity of their corresponding drfp fingerprints. (c) During fine-tuning, the model receives reaction pairs along with the Tanimoto score of their drfp fingerprints as targets. The mean squared error (MSE) between the predicted cosine similarity and the drfp-derived similarity is minimized.

Our approach focuses on a chemical reaction similarity-based strategy for enzyme prediction, which offers high interpretability and straightforward validation. Central to this approach is the vectorized molecular representations, known as reaction fingerprints, that capture the essence of chemical transformations. These fingerprints are essential in cheminformatics applications such as reaction classification, similarity search, and synthesis planning (Schneider *et al.* 2015, Baylon *et al.* 2019, Shen *et al.* 2021). An ideal reaction fingerprint should encode the core transformation patterns of a reaction and enable similar reactions to cluster naturally in the latent space, which is particularly important when using similarity-based methods to predict potential enzymes based on existing enzyme-reaction associations. Traditional fingerprints, such as reaction difference fingerprints based on hashed molecular descriptors (e.g. Morgan, Atom Pair, and Topological Torsion) (Morgan 1965, Pérez-Nueno *et al.* 2009, Schulz-Gasch *et al.* 2012), encode atom and bond connectivity and highlight structural differences between reactants and products. One such example is the differential reaction fingerprint (drfp), which computes the symmetric difference between circular fingerprints of reactants and products (Probst *et al.* 2022b). However, despite being simple and efficient, these methods can suffer from bit collisions due to hashing, especially when compressed into short vectors. Consequently, such fingerprints need to be long enough to alleviate bit collisions. However, using high-dimensional input features on small datasets increases the risk of overfitting and limits the generalizability of machine learning models in small-data scenarios such as environmental applications. Meanwhile, recent advances in deep learning have led to the development of data-driven alternatives that showed potential to capture the critical features of chemical transformations (Schwaller *et al.* 2021b, Wen *et al.* 2022, Li *et al.* 2023). For example, rxnfp fingerprints uses a BERT-style transformer model pre-trained on large reaction SMILES corpora via masked language modeling to produce 256-dimensional vector representations, which showed strong performance in various reaction-centric tasks (Schwaller *et al.* 2021b). However, without task-specific fine-tuning, rxnfp fingerprints sometimes fail to cluster reactions by transformation type, limiting their applicability in similarity-based tasks. Recent contrastive learning frameworks, including RxnRep and RxnGraph, aim to solve this issue by training models to group reactions with similar transformations and push apart unrelated reactions (Wen *et al.* 2022, Li *et al.* 2023). Nevertheless, many of these methods require curated reaction annotations such as atom-mapping or reaction center information for training. While some of these annotations can be generated automatically using computational tools (Schwaller *et al.* 2021a), the outputs may not generalize well across all reaction types due to the inherent combinatorial complexity of atom-mapping, especially for reactions involving a large number of atoms and complex rearrangements. In contrast, no such annotations are required in our similarity-based strategy. In this work, as shown in Fig. 1, we propose a scalable and data-efficient fine-tuning strategy for rxnfp, designed to align its learnt representations with the structural transformation patterns encoded by drfp, which reflects the molecular changes that occur between reactants and products. Specifically, we use the Tanimoto similarity between drfp fingerprints as a supervisory signal to train rxnfp embeddings such that reactions with high drfp similarity also show high cosine similarity in the rxnfp space. The proposed contrastive, self-supervised approach leverages algorithmically generated similarity scores without relying on expert annotations. The resulting 256-dimensional fingerprints, which we refer to as reaction center-specific fingerprints (crxnfp), effectively represent reaction-specific transformation features, enabling reactions with similar transformations to cluster naturally in the embedding space and making them well-suited for similarity-based applications. Additionally, the compact size of these fingerprints supports efficient learning in domains with limited training data, such as environmental studies.

## 2 Materials and methods

### 2.1 Data curation

**Figure 2:**
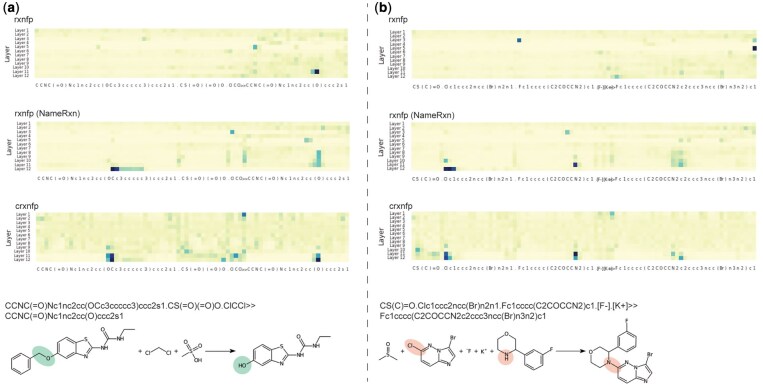
Attention visualization of BERT models for reaction fingerprint generation. Layer-wise attention maps are shown for BERT models producing rxnfp, rxnfp (NameRxn), and crxnfp fingerprints. For rxnfp and rxnfp (NameRxn), attention scores were derived from the [CLS] token. For crxnfp, which used mean-pooled token embeddings, attention scores were averaged across all tokens. The horizontal axis represents SMILES tokens of the input reaction, while the vertical axis corresponds to the transformer layers. Darker shading indicates higher attention weights for a given token at a specific layer. Reaction centers in both substrates and products are highlighted.

Our model is a fine-tuned version of a transformer-based architecture (i.e. BERT) (Devlin *et al.* 2019) originally designed to generate rxnfp fingerprints (Schwaller *et al.* 2021b). The initial pre-training was conducted on 2.6 million reactions extracted from the Pistachio database (version 191118) (https://www.nextmovesoftware.com/pistachio), after removing duplicates and invalid entries. During pre-training, a masked language modeling task was employed with a masking probability of 15%. For fine-tuning, we used a dataset of 2.8 million reactions previously compiled from both grants and patent applications. This dataset was preprocessed by removing the original atom-mapping, canonicalizing molecular structures using RDKit, and eliminating duplicate reactions. From this cleaned dataset, we sampled 1 million pairs of reactions. For each reaction pair, we computed the Tanimoto similarity score using drfp fingerprints. As the majority of these similarity scores were between 0 and 0.3, we applied a transformation defined in Equation (1) to shift the distribution rightward while keeping the transformed values still within 0–1. We selected the coefficient of 5 for the transformation as a practical compromise based on the observed distribution of reaction Tanimoto similarities. This choice provides a meaningful rightward shift of the similarity distribution, improving resolution among reaction pairs with low to moderate similarity, while avoiding overly rapid saturation of the transformed score toward 1.0. The transformed Tanimoto scores served as target labels during fine-tuning. The distributions of Tanimoto scores before and after transformation can be found in Figs 1 and 2, available as supplementary data at *Bioinformatics* online


(1) Transformed score = 1 - exp (-5 × Tanimoto)

To ensure a uniform distribution of Tanimoto scores across the target range (i.e. 0–1) for training, we partitioned this interval into seven bins, namely, 0–0.1, 0.1–0.2, 0.2–0.3, 0.3–0.4, 0.4–0.5, 0.5–0.6, 0.6–1.0. For the first six bins, we sampled 0.1 million reaction pairs per bin, ensuring that their transformed scores fell within the corresponding ranges. However, since reaction pairs with high similarities were relatively rare, the last bin had a broader range (i.e. 0.6–1.0) than the others, and 0.4 million reaction pairs were sampled into this bin. The distribution of the Tanimoto scores of training samples can be found in Fig. 2b, available as supplementary data at *Bioinformatics* online. The fine-tuning progress of models on the training samples is shown in Section 8, available as supplementary data at *Bioinformatics* online. Figure 5, available as supplementary data at *Bioinformatics* online, shows the distribution of cosine similarities between crxnfp fingerprints calculated for 1000 pairs of forward and reverse reactions, which was used to assess the influence of reaction directionality on the learned embeddings.

### 2.2 Reaction classification

To evaluate the performance of crxnfp representations in reaction classification tasks, we selected three datasets of varying sizes. First, we employed an open-source dataset, USPTO 1k TPL, derived from the USPTO database (https://doi.org/10.6084/m9.figshare.5104873). It contained 445 000 reactions categorized into 1000 reaction templates using a template extraction workflow established in previous studies (Schwaller *et al.* 2021b). The dataset was divided only once into 90% for training and validation and 10% for testing due to its large size, and we adopted the same split as in previous studies (Probst *et al.* 2022b). Second, we collected reactions from the EAWAG-BBD and EAWAG-SOIL data packages available in enviPath (Latino *et al.* 2017). The association of EAWAG-BBD and EAWAG-SOIL biotransformation rules with their corresponding functional enzymes and protein sequences was achieved by retrieving cross-references as is further described in Section 7, available as supplementary data at *Bioinformatics* online. Reactions were grouped using enviRule based on reaction centers as well as the atoms and bonds altered during the reactions. We kept only reaction groups with more than five reactions, resulting in 67 reaction groups and a total of 1029 reactions, with the group numbers serving as classification labels. Similarly, this dataset was split into 90% for training and validation and 10% for testing, and we performed the random split 10 times for a stable evaluation. Additionally, we tested on Schneider 50k dataset, which contains 50 000 reactions evenly distributed among 50 reaction classes as annotated by the NameRxn tool (https://www.nextmovesoftware.com/namerxn.html). In accordance with previous studies (Probst *et al.* 2022b), 10 000 reactions were randomly selected for training and validation, while the remaining 40 000 reactions were used for testing, repeated for 10 times. Additionally, we applied the crxnfp-based enzyme prediction method to a new experimental dataset primarily composed of biotransformation reactions involving fluorinated micropollutants as described in Section 6, available as supplementary data at *Bioinformatics* online.

### 2.3 Model training for reaction center-specific fingerprints

Similar to the BERT model used to generate rxnfp fingerprints, our model consisted of 12 transformer layers. The hidden size for both the embeddings and the output of each transformer layer was 256, while the intermediate size of the feed-forward network (FFN) within each transformer layer was 512. Each transformer layer contained four attention heads. In contrast to the original BERT model for rxnfp, we introduced an additional pooling layer that averaged the embeddings of all tokens. The resulting pooled embeddings, referred to as crxnfp fingerprints, were used to compute the cosine similarity between reaction pairs. The model was fine-tuned by minimizing the mean squared error (MSE) between the cosine similarity of crxnfp fingerprints and the transformed Tanimoto scores of drfp fingerprints. For comparison, we also fine-tuned another model that directly used the embeddings from the first token (i.e. [CLS]) as crxnfp fingerprints, which was consistent with the original BERT model generating rxnfp fingerprints. During fine-tuning, the maximum learning rate was set to 5 × 10^−5^, with 10% of the total training steps allocated for warm-up, and the model was trained for five epochs with a batch size of 32. Early stopping was employed, terminating training if no improvement in validation performance was observed for 200 consecutive steps. The development and fine-tuning of models were conducted using the Sentence Transformers (version 3.4.0.dev0) Python module maintained by Hugging Face (Reimers *et al.* 2019). The final model was converted into a PyTorch format to facilitate further analysis. The details of *K*-nearest-neighbor (KNN) classifiers and multi-layer perceptron (MLP) models for reaction classification can be found in SI Sections 1.3 and Section 1.4, available as supplementary data at *Bioinformatics* online, respectively.

## 3 Results

### 3.1 Reaction classification results

The performance of reaction classification was evaluated using three different datasets of varying sizes, namely, USPTO 1K TPL, EAWAG-BBD/SOIL reactions, and Schneider 50k dataset. To ensure comparability with previously reported performance, we conducted reaction classification using MLP models and FAISS-assisted KNN classifiers (Johnson *et al.* 2021). The classification was performed using multiple reaction fingerprints, including rxnfp, crxnfp, drfp, and rdkit fingerprints. Due to the inefficiency of k-NN models when handling high-dimensional fingerprints, we restricted their use to fingerprints of relatively manageable length, including rxnfp (256 bits), crxnfp (256 bits), and drfp (2048 bits). On the contrary, rdkit fingerprints, which contained 4096 bits, were only evaluated using MLP models to accommodate their high dimensionality.

As shown in Table 1, for the USPTO 1K TPL dataset, the 5-NN model trained with rxnfp fingerprints only achieved a classification accuracy of 0.34. In contrast, the 5-NN model trained with crxnfp fingerprints showed a substantial improvement, achieving an accuracy of 0.90, which was comparable to the 5-NN model trained with drfp fingerprints showing an accuracy of 0.92. Additionally, the MLP trained with crxnfp reached 98% of the performance of the 5-NN model trained with fine-tuned version of rxnfp (i.e. BERT-CLS in Table 1), which were specifically fine-tuned on the USPTO 1K TPL dataset in a BERT model. For the EAWAG-BBD/SOIL reactions, we compared the prediction performance of 3-NN models with rxnfp, crxnfp, drfp fingerprints. In addition, we included a 3-NN model with rxnfp (NameRxn) fingerprints, another fine-tuned variant of rxnfp fingerprints obtained by fine-tuning rxnfp on 2.6 million reactions extracted from the proprietary Pistachio database. As reported in the previous study (Schwaller *et al.* 2021b), each reaction in Pistachio database was classified using the rule-based software NameRxn, which assigned reaction classes based on RXNO ontology, enabling the distinction of reactions with different transformation mechanisms (Probst and Reymond 2020). Consequently, the rxnfp (NameRxn) fingerprints should be sufficiently informed to effectively capture structural transformations in reactions. As both the Pistachio database and NameRxn are proprietary resources, we did not perform fine-tuning ourselves but instead directly utilized the trained rxnfp (NameRxn) from the previous work to generate reaction fingerprints (Schwaller *et al.* 2021b), which were then used in a 3-NN model. As shown in Table 1, the 3-NN model trained with crxnfp fingerprints outperformed all other 3-NN models. It achieved an accuracy of 0.86, representing a 46% improvement over the 3-NN models trained with rxnfp and drfp fingerprints. Notably, even when compared to the 3-NN model trained with rxnfp (NameRxn) fingerprints, which were specifically fine-tuned for transformation classification, the crxnfp-based model still showed a 5% improvement in accuracy. For the Schneider 50k dataset, the MLP model trained with crxnfp fingerprints outperformed all other MLP models trained with rxnfp, drfp, and rdkit fingerprints. In particular, the crxnfp-based MLP achieved an improvement in accuracy of more than 10% over the rxnfp-based MLP, as shown in Table 1.

**Table 1:** Reaction classification performance with different reaction fingerprints.^a^

Fingerprints	Classifier	Accuracy	CEN	MCC
USPTO 1k TPL				
rxnfp	5-NN	0.340	0.392	0.337
crxnfp	5-NN	0.900	0.050	0.900
drfp	5-NN	0.917	0.041	0.917
rxnfp	BERT-CLS	**0.989**	**0.006**	**0.989**
*crxnfp*	*MLP*	*0.969*	*0.014*	*0.969*
drfp	MLP	0.977	0.011	0.977
EAWAG-BBD/SOIL				
rxnfp	3-NN	0.589 ± 0.028	0.082 ± 0.006	0.579 ± 0.026
rxnfp (NameRxn)	3-NN	0.829 ± 0.014	0.031 ± 0.004	0.825 ± 0.014
*crxnfp*	*3-NN*	***0.860 ± 0.010***	***0.024 ± 0.003***	***0.857 ± 0.011***
drfp	3-NN	0.589 ± 0.022	0.073 ± 0.006	0.582 ± 0.020
Schneider 50k				
rxnfp	MLP	0.873 ± 0.009	0.119 ± 0.004	0.870 ± 0.009
*crxnfp*	*MLP*	***0.966 ± 0.002***	***0.041 ± 0.001***	***0.965 ± 0.002***
drfp	MLP	0.955 ± 0.004	0.056 ± 0.002	0.954 ± 0.004
rdkit	MLP	0.857 ± 0.004	0.130 ± 0.004	0.855 ± 0.004

a The calculations of conditional entropy (CEN) and Matthews correlation coefficient (MCC) are defined in Section 1, available as supplementary data at *Bioinformatics* online. The new method reported here (crxnfp) is highlighted in gray italics. Best performance for each data set is highlighted in bold.

### 3.2 Visualization of attention

Figure 2 presents the layer-wise attention of the [CLS] token for the BERT models generating rxnfp and rxnfp (NameRxn) fingerprints, as these models retained only the embedding of the [CLS] token. Additionally, Fig. 2 also shows the layer-wise averaged attention across all tokens for the BERT model generating crxnfp fingerprints, since crxnfp fingerprints were derived by averaging the embeddings of all tokens rather than relying solely on the [CLS] token. Our analysis of the attention weights revealed that, without task-specific fine-tuning, the BERT model producing rxnfp fingerprints could not effectively focus on reaction centers. Specifically, the model either highlighted incomplete reaction centers or completely missed them, which could explain its suboptimal performance in reaction classification. In contrast, the BERT models generating rxnfp (NameRxn) and crxnfp fingerprints successfully focused on reaction centers, though with some variations in their attention patterns. In the first example shown in Fig. 2a, the reaction involved the cleavage of a C–O bond, leading to the formation of a hydroxyl group. As a result, the reaction centers in the substrates should include a carbon and an oxygen atom, while in the products, the reaction centers should include the hydroxyl group. The BERT model generating rxnfp fingerprints primarily focused on the hydroxyl group in the products but failed to attend to the reaction centers in substrates. Conversely, the BERT model generating rxnfp (NameRxn) successfully identified the reaction centers in the products. However, in the substrates, instead of focusing on the oxygen atom, it attended to the benzene ring connected to the carbon atom in the reaction center. On the contrary, the BERT model generating crxnfp correctly highlighted the reaction centers in both the substrates and the products. In the second example shown in Fig. 2b, an aromatic chloride was replaced by a nitrogen from a morpholine ring derivative. Accordingly, the reaction center in substrates should include the aromatic chloride and the adjacent aromatic carbon atom, as well as the nitrogen atom from the morpholine derivative. Similarly, in the products, the reaction center should consist of the aromatic carbon atom and the morpholine nitrogen. The attention analysis indicated that the BERT model generating rxnfp fingerprints failed to focus on the relevant reaction centers, instead incorrectly attending to the fluorine atom in both the substrates and the products. Although the BERT model generating rxnfp (NameRxn) successfully identified reaction centers in both the substrates and the products, it also attended to the numerical ring indices in the SMILES representation, e.g. “1” in the substrates and “2” in the products. These numerical indices were just syntactic components of the SMILES notation and were not chemically relevant to reaction centers. Notably, they were ignored by the BERT model generating crxnfp fingerprints, which focused exclusively on chemically meaningful reaction centers. Furthermore, we observed a distinct attention pattern in the BERT model generating crxnfp fingerprints. The initial layers predominantly attended to the separator between substrates and products in SMILES notation (i.e. “≫”) while exhibiting weak attention to other tokens. As the depth of the model increased, the attention progressively shifted toward the reaction centers in the substrates and products.

In this attention analysis, the BERT generating crxnfp fingerprints showed comparable or even superior attention to reaction centers compared to the BERT generating rxnfp (NameRxn) fingerprints. Unlike the BERT generating rxnfp (NameRxn), the BERT model generating crxnfp fingerprints was not explicitly fine-tuned to classify reactions based on distinct transformation. Nevertheless, the contrastive fine-tuning approach employed to train BERT model for generating crxnfp fingerprints effectively captured reaction centers, as supported by the attention analysis. The ability of crxnfp fingerprints to focus on reaction centers suggests that, though not being explicitly designed for reaction mechanism classification, the contrastive fine-tuning process enabled the model to learn and emphasize the most critical structural features that distinguish different transformation reactions.

### 3.3 Association of enviPath rules and reactions with UniProt enzymes

**Figure 3:**
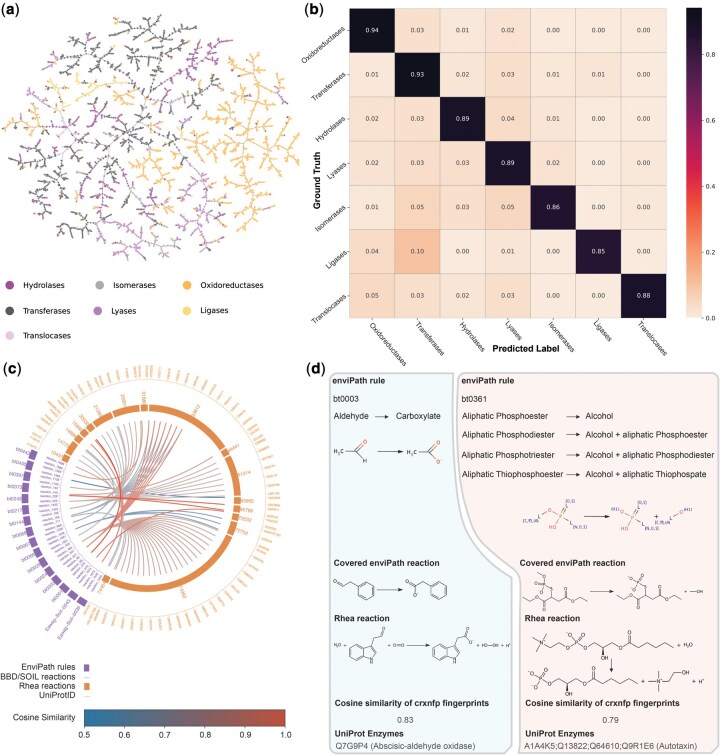
Evaluation and application of crxnfp fingerprints. (a) TMAP visualization constructed from crxnfp fingerprints shows that reactions naturally cluster according to their respective EC superclasses. (b) Per-class classification accuracy of an MLP model trained on crxnfp fingerprints shows high performance across most superclasses. (c) Schematic of the enzyme association workflow: for each BBD/SOIL reaction covered by an enviPath rule, the most similar Rhea reactions were identified based on cosine similarity of crxnfp fingerprints (threshold > 0.5). As Rhea reactions are linked to UniProt enzymes, it enables the indirect association of biotransformation rules/reactions with specific UniProt enzymes. (d) Two representative examples of biotransformation rule/reaction associations with UniProt enzymes, highlighting that highly similar Rhea reactions can be retrieved through crxnfp-based similarity search.

To validate the feasibility of associating enviPath biotransformation rules or reactions with UniProt enzymes based on the cosine similarity of crxnfp fingerprints, we first assessed whether crxnfp can effectively classify reactions according to their underlying transformation mechanisms. As a benchmark, we used the Rhea database, in which each reaction was annotated with one of the seven Enzyme Commission (EC) superclasses, namely, Oxidoreductases, Transferases, Hydrolases, Lyases, Isomerases, Ligases, and Translocases. The distribution across classes was imbalanced, with Oxidoreductases comprising 34.5% of the dataset and Translocases only 1.1%. We computed the crxnfp fingerprints for all 7575 Rhea reactions and visualized their similarity using a TMAP (Probst and Reymond 2020), as shown in Fig. 3a, where each point was colored according to its EC superclass. Notably, despite no fine-tuning on EC-labeled datasets had taken place, the crxnfp fingerprints naturally formed well-separated clusters corresponding to the seven EC classes as shown in Fig. 3a. Moreover, subtrees in the TMAP mostly contained reactions from the same EC superclass, suggesting that crxnfp is capable of distinguishing reactions at a finer-grained level.

To further quantify this ability, we trained a simple MLP classifier with one hidden layer of 512 nodes to predict the EC superclass of Rhea reactions from their crxnfp fingerprints. The model was trained for 20 epochs using an 80/20 random train-test split, and the experiment was repeated 10 times. The classifier achieved an average accuracy of 0.912 ± 0.003 (CEN = 0.150 ± 0.004, MCC = 0.903 ± 0.003), showing high predictive performance. The confusion matrix in Fig. 3b suggested that this accuracy was consistent across most EC classes. These findings support that a crxnfp-based search is capable of identifying Rhea reactions similar to biotransformation reactions in enviPath. Two representative examples are shown in Fig. 3d and the results are further discussed in Section 7, available as supplementary data at *Bioinformatics* online.

Above examples elucidate the effectiveness of using crxnfp-based similarity to connect transformation rules with relevant enzymes. The ability to associate enviPath rules with UniProt enzymes opens the possibility to the automatic annotation of reaction patterns and enzymatic information in large-scale reaction databases. In our previous work, we introduced enviRule, a tool for automated extraction of biotransformation rules from large reaction datasets (Zhang and Fenner 2023). However, the enzymes responsible for catalyzing these reactions remained largely unknown. The similarity-based association method proposed here offers a promising solution to bridge this gap. Furthermore, the similarity search algorithm in this work is highly efficient. For each of the 1769 biotransformation reactions, we identified the five most similar reactions from the 7575 Rhea reactions using cosine similarity of crxnfp fingerprints, which required only 0.117 s on a Mac mini (2018) equipped with a 3.2 GHz 6-core Intel Core i7 processor, showing the scalability of our approach for large datasets.

### 3.4 Enzyme class classification of biotransformation reactions involving fluorinated compounds

To evaluate its performance, the proposed method is benchmarked against two state-of-the-art classification-based tools, namely, Theia (Probst 2023) and CLAIRE (Zeng *et al.* 2025). Both tools formulate enzyme prediction as a supervised multi-class classification problem, directly learning to map reactions to EC numbers using MLP. Theia employs a relatively simple architecture, consisting of a single hidden layer MLP trained on drfp, which represent the symmetric difference in molecular features between reactants and products. In contrast, CLAIRE adopts a deeper architecture with five hidden layers and leverages a contrastive training objective, which is conceptually similar to the approach used in training crxnfp fingerprints. Both Theia and CLAIRE have shown strong generalization capabilities and competitive performance on the ECREACT dataset, a large, curated dataset containing over 80 000 EC-annotated reactions (Probst *et al.* 2022a).

Table 2 summarizes the prediction accuracy for EC number classification at levels 1, 2, and 3 using crxnfp, Theia, and CLAIRE. The crxnfp-based method demonstrates consistently high performance, achieving 91.30% accuracy across all EC levels. This result indicates strong reliability of this method in predicting enzymes for the biotransformation of contaminants. Theia shows moderate predictive ability, with accuracies of 56.52% at level 1 and 34.78% at both levels 2 and 3. In contrast, CLAIRE exhibits limited performance on this dataset, reaching only 13.04% accuracy at level 1 and 4.34% at levels 2 and 3. The detailed prediction results for each reaction can be found in Table 3, and the visualizations of reactions are shown in Table 1, available as supplementary data at *Bioinformatics* online. Of note, the crxnfp approach could not handle predictions for N,N-diethyl-3-methylbenzamide (DEET) and its analog N,N-dimethyl 4-chlorobenzamide, as no sufficiently similar reactions were found in the Rhea database using a cosine similarity threshold of 0.5. By contrast, other approaches did return predictions for the corresponding EC. However, only Theia made a correct prediction at the first level.

**Table 2:** Prediction accuracy of crxnfp, Theia, and CLAIRE for enzyme classification at EC levels 1, 2, and 3 on the curated dataset of fluorinated compound biotransformation reactions.

	Level 1	Level 2	Level 3
CLAIRE	13.04%	4.34%	4.34%
Theia	56.52%	34.78%	34.78%
crxnfp	**91.30%**	**91.30%**	**91.30%**

Best performance for each level is highlighted in bold.

**Table 3:** Detailed enzyme prediction results for individual reactions using CLAIRE, Theia, and crxnfp.

Reaction	EC Class	CLAIRE Pred	Theia Pred	crxnfp Pred	Reference
5-Fluorouracil + NADPH → 5,6-dihydrofluorouracil + NADP^+^	1.3.1.2	1.16.1	1.3.1	1.3.1.2	Spanogiannopoulos *et al.* (2022)
5,6-Dihydrofluorouracil + H_2_O → 2-fluoro-3-ureidopropionic acid + H^+^	3.5.2.2	1.3.7	3.5.2	3.5.2.2	This study (Table 2, available as supplementary data at *Bioinformatics* online; two new enzymes)^a^
2-Fluoro-3-ureidopropionic acid + H_2_O + H^+^ → 3-amino-2-fluoropropanoic acid + ${\mathrm{NH}}_{4}^{+}$ + CO_2_	3.5.1.6	5.1.3	3.5.1	3.5.1.6	Hishinuma *et al.* (2020)
3-Amino-2-fluoropropanoic acid + H_2_O → 3-aminopropanoic acid + H^+^ + F^−^	3.8.1.2	3.5.1	3.2.1	3.8.1.3	Probst et al. (2025)
Fluoroacetate + H_2_O → glycolate + H^+^ + F^−^	3.8.1.3 AND 3.8.1.2	1.1.1	3.8.1	3.8.1.3	Probst *et al.* (2025)
Difluoroacetate + H_2_O → 2-fluoro-2-hydroxyacetate + H^+^ + F^−^	3.8.1.3 AND 3.8.1.2	1.1.1	3.1.1	3.8.1.3	Probst *et al.* (2025)
(*E*)-2,3,5,5,5-pentafluoro-4-(trifluoromethyl)pent-2-enoic acid + ATP + CoA → (*E*)-2,3,5,5,5-pentafluoro-4-(trifluoromethyl)pent-2-enoic acid CoA + AMP + diphosphate	6.2.1	1.16.1	2.3.1	6.2.1.-	Yu *et al.* (2024b)
*(E)*-2,3,4,4,5,5,6,6,6-nonafluorohex-2-enoic acid + ATP + CoA → (*E*)-2,3,4,4,5,5,6,6,6-nonafluorohex-2-enoic acid CoA + AMP + diphosphate	6.2.1	1.16.1	2.3.1	6.2.1.-	Yu *et al.* (2024b)
(*E*)-2,3,4,4,5,5,6,6,7,7,8,8,8-tridecafluorooct-2-enoic acid + ATP + CoA → (*E*)-2,3,4,4,5,5,6,6,7,7,8,8,8-tridecafluorooct-2-enoic acid CoA + AMP + diphosphate	6.2.1	1.16.1	2.3.1	6.2.1.-	Yu *et al.* (2024b)
(*E*)-caffeate + ATP + CoA → (*E*)-caffeoyl-CoA + AMP + diphosphate	6.2.1	2.8.2	6.2.1	6.2.1.-	Yu *et al.* (2024b)
(*E*)-caffeoyl-CoA + oxidized [2Fe-2S]-[ferredoxin] + NADH → hydrocaffeoyl-CoA + reduced [2Fe-2S]-[ferredoxin] + NAD^+^	1.3.1.108	5.1.3	1.3.1	1.3.1.108	Yu *et al.* (2024b)
5,5,6,6,6-Pentafluorohexanoic acid + ATP + CoA → 5,5,6,6,6-pentafluorohexanoic acid CoA + AMP + diphosphate	6.2.1	1.16.1	2.3.1	6.2.1.40	Rivera‐Cancel et al. (2012)
3,3,3-Trifluoropropanoic acid + ATP + CoA → 3,3,3-trifluoropropanoic acid CoA + AMP + diphosphate	6.2.1	5.1.3	6.2.1	6.2.1.40	Mothersole *et al.* (2024)
5,5,5-Trifluoropentanoic acid + ATP + CoA → 5,5,5-trifluoropentanoic acid CoA + AMP + diphosphate	6.2.1	2.1.1	2.3.1	6.2.1.40	Mothersole *et al.* (2024)
4,5,5-Trifluoropent-4-enoic acid + ATP + CoA → 4,5,5-trifluoropent-4-enoic acid CoA + AMP + diphosphate	6.2.1	1.16.1	2.3.1	6.2.1.40	Mothersole *et al.* (2024)
4,4,5,5,5-Pentafluoropentanoic acid + ATP + CoA → 4,4,5,5,5-pentafluoropentanoic acid CoA + AMP + diphosphate	6.2.1	1.16.1	2.3.1	6.2.1.40	Mothersole *et al.* (2024)
(*E*)-2,3,5,5,5-pentafluoro-4-(trifluoromethyl)pent-2-enoic acid CoA + oxidized [2Fe-2S]-[ferredoxin] + NADH → 2,3,5,5,5-pentafluoro-4-(trifluoromethyl)pentanoic acid CoA + reduced [2Fe-2S]-[ferredoxin] + NAD^+^	1.3.1.108	5.1.3	1.18.1	1.3.1.108	Yu *et al.* (2024b)
(*E*)-2,3,4,4,5,5,6,6,6-nonafluorohex-2-enoic acid CoA + oxidized [2Fe-2S]-[ferredoxin] + NADH → 2,3,4,4,5,5,6,6,6-nonafluorohexanoic acid CoA + reduced [2Fe-2S]-[ferredoxin] + NAD^+^	1.3.1.108	5.1.3	**Fail**	1.3.1.108	Yu *et al.* (2024b)
(*E*)-4,6,6,6-tetrafluoro-5-(trifluoromethyl)hex-2-enoic acid CoA + oxidized [2Fe-2S]-[ferredoxin] + NADH → 4,6,6,6-tetrafluoro-5-(trifluoromethyl)hexanoic acid CoA + reduced [2Fe-2S]-[ferredoxin] + NAD^+^	1.3.1.108	1.3.1	**Fail**	1.3.1.108	Yu *et al.* (2024b)
(*E)*-2,3,4,4,5,5,6,6,7,7,8,8,8-tridecafluorooct-2-enoic acid CoA + oxidized [2Fe-2S]-[ferredoxin] + NADH → 2,3,4,4,5,5,6,6,7,7,8,8,8-tridecafluorooctanoic acid CoA + reduced [2Fe-2S]-[ferredoxin] + NAD^+^	1.3.1.108	5.1.3	**Fail**	1.3.1.108	Rivera-Cancel et al. (2012)
Paracetamol + H_2_O → 4-aminophenol + H^+^ + acetate	3.5.1.13	2.1.1	3.5.1	3.5.1.135	Rios-Miguel et al. (2022)
*N,N*-Diethyl-3-methylbenzamide (DEET) + H_2_O → 3-methylbenzoic acid + H^+^ + diethylamine	3.5.1	6.3.4	3.1.1	No analogous reactions in Rhea	Rivera-Cancel et al. (2012)
*N,N*-Dimethyl 4-chlorobenzamide + H_2_O → 4-chlorobenzoic acid + H^+^ + dimethylamine	3.5.1	2.1.1	3.1.1	No analogous reactions in Rhea	Yu *et al.* (2024a)

a Microbial enzymes newly characterized for this study are described in Table 2, available as supplementary data at *Bioinformatics* online.

The failed cases are highlighted in bold.

This performance is particularly notable given that, unlike Theia and CLAIRE, the crxnfp approach was not trained on a dataset labeled with EC numbers. Instead, the model generating crxnfp was trained using a contrastive learning framework to capture the similarity of chemical transformation between pairs of reactions. Although crxnfp was not trained with EC-specific supervision, it still outperforms both Theia and CLAIRE, which are explicitly trained on ECREACT. The observed performance gap highlights potential limitations of supervised models when applied to underrepresented chemical spaces. While both Theia and CLAIRE report strong generalization performance on ECREACT, with published EC level 3 accuracies exceeding 95%, their performance drops significantly on reactions involving fluorinated compounds. One likely explanation is the limited representation of fluorinated compounds in the ECREACT dataset, where only 230 out of 81 205 reactions involve fluorinated compounds. This imbalance limits the ability of models to generalize across the chemical space of fluorinated compounds, even though they perform well on more commonly represented biochemical reactions. Notably, crxnfp was able to accurately predict the EC numbers and corresponding enzymes for transformation products (TPs) of fluorinated compounds. In agreement with crxnfp results, we experimentally verified two new enzymes catalyzing reactions of fluorinated compounds in this study, detailed in Section 6, available as supplementary data at *Bioinformatics* online, highlighting a use case for crxnfp to identify enzyme candidates.

An additional aspect of the evaluation focuses on comparing the performance of CLAIRE and crxnfp, both of which employ contrastive learning strategies within their training frameworks. CLAIRE was designed to distinguish enzymatic classes through contrastive training based on Triplet Margin Loss, which encourages reactions within the same EC class to be embedded closer in the learned feature space, while reactions from different classes are pushed farther apart. However, this training method enforces binary similarity relationships based solely on whether two reactions belong to the same EC class, without capturing finer transformation similarities that may exist within or across EC classes. Consequently, reactions in the training dataset sharing an EC number but differing in their underlying transformation may be embedded too closely, potentially leading to overfitting. In contrast, crxnfp embeddings are trained to reproduce continuous transformation similarity between reactions. The contrastive training approach for crxnfp captures fine-graded similarities across all reaction pairs, allowing the model to capture varying degrees of similarity within each EC class, which enables crxnfp to provide more informative and discriminative representations in similarity-based enzyme prediction. The comparison of prediction performance has shown that, though not explicitly trained on EC-labeled data, the similarity search using crxnfp offers a robust and generalizable strategy for enzyme prediction across challenging chemical spaces (e.g. fluorinated compounds).

However, the failure of the crxnfp method to generate enzyme predictions for DEET and its analog highlights an important limitation of similarity-based approaches. These methods depend on the presence of structurally related reactions in the reference database. When no reaction reaches the defined similarity threshold, set at a cosine similarity of 0.5 in this study, the model is unable to produce a prediction. This reveals a constraint in applying such models to novel reactions, particularly when they fall outside the transformation space represented in databases such as Rhea. Future research could investigate the use of adaptive similarity thresholds that consider the distribution of reactions in the embedding space. Another direction could involve combining similarity-based inference with supervised learning models to improve prediction coverage in sparsely populated regions of chemical space. Additionally, expanding curated reaction databases to include a wider range of environmentally relevant and emerging contaminants would also improve the robustness and applicability of enzyme prediction frameworks such as crxnfp.

## 4 Conclusion

In this study, we introduced a self-supervised, contrastive fine-tuning strategy for the rxnfp reaction fingerprint model, aligning its learnt representations with transformation-specific similarities encoded in drfp fingerprints. Our approach enhanced the consistency between transformation patterns and the latent space of reaction embeddings, thereby enabling chemically meaningful clustering and similarity-based applications. Among the two pooling strategies evaluated, mean-pooled embeddings outperformed [CLS]-based representations, offering better training stability and validation performance. The resulting crxnfp fingerprints showed excellent capability to differentiate reactions by transformations, supported by various reaction datasets, and learned strong attention to chemically relevant reaction centers. Although not being fine-tuned on proprietary datasets or annotated reaction transformations, the contrastively fine-tuned model learned to encode transformation information in an interpretable and scalable way.

A key application of crxnfp fingerprints is their ability to improve the enzymatic annotation of contaminant biotransformation databases such as enviPath by predicting enzyme associations for unannotated environmental biotransformation reactions based on the cosine similarity of crxnfp fingerprints to those of curated enzyme-associated reactions from databases such as Rhea. It opens the possibility to the automated enrichment of large-scale environmental reaction datasets with enzymatic information, which is essential for mechanistic insight, compound prioritization, and the rational design of biotransformation-informed treatment systems. More broadly, crxnfp fingerprints carry important implications for environmental modeling. The compact 256-dimensional fingerprints are especially well-suited for modeling in data-limited settings, which is a common challenge in environmental study. Additionally, our approach also offers the potential to refine TP predictions by filtering out reactions that are not plausible given the enzymatic composition in a specific environment, making prediction results more targeted and representative of actual environmental scenarios. Together, these capabilities facilitate improved understanding of contaminant fate, support data-driven risk assessment, and contribute to the development of enzyme-targeted contaminant management strategies across diverse environmental settings.

## Supplementary Material

btag142_Supplementary_Data

## Data Availability

The scripts used to generate reaction pairs are publicly available at https://github.com/zhangky12/crxnfp_sample_prep. The repository https://github.com/zhangky12/crxnfp contains the code for fine-tuning BERT-based models and generating crxnfp fingerprints. The implementation for similarity-based chemical reaction search using crxnfp fingerprints is provided at https://github.com/zhangky12/crxnfp_knn. Pre-computed fingerprints (crxnfp, rxnfp, and drfp) can be accessed and downloaded from https://zenodo.org/records/16996192.
