# Supplementary material for: Enzyme association for environmental biotransformation reactions through contrastive learning of reaction center-specific fingerprints
Source: Bioinformatics. 2026 Mar 24;42(4):btag142. doi: 10.1093/bioinformatics/btag142 (PMC13091653; doi:10.1093/bioinformatics/btag142)
Supplement: btag142_Supplementary_Data [file btag142_supplementary_data.pdf]

Enzyme Association for Environmental Biotransformation Reactions Through Contrastive Learning of Reaction-Center-Specific Fingerprints

Kunyang Zhang[1,2,*], Thierry D. Marti[3,4], Silke I. Probst[3], Serina L. Robinson[3,4], Kathrin Fenner[1,2]

[1]Department of Environmental Chemistry, Eawag, 8600 Dübendorf, Switzerland

[2]Department of Chemistry, University of Zürich, 8057 Zürich, Switzerland

[3]Department of Environmental Microbiology, Eawag, 8600 Dübendorf, Switzerland

[4]Department of Environmental Systems Science, ETH Zurich, 8057 Zürich, Switzerland

Table of Contents

1. Evaluation metrics and classification models 2

1.1 Conditional Entropy (CEN) 2

1.2 Matthews Correlation Coefficient (MCC) 2

1.3 The K-Nearest-Neighbor Classifier 3

1.4 Multi-Layer Perceptron for Reaction Classification 3

2. Methodology for data visualization 3

2.1 TMAP 3

2.2 Attention visualization 5

3. Distribution of Tanimoto scores before and after transformation 6

4. Model explainability through extra attention analysis 7

5. Exemplar training reaction pairs with transformed Tanimoto scores 21

6. Experimental validation of fluorinated compound biotransformation reactions 26

6.1 Experimental dataset curation 26

6.2 Cloning, expression and protein purification 30

6.3 Enzyme activity assays 31

7. Association of enviPath rules and reactions with UniProt Enzymes 35

8. Fine-tuning progress of large language models 35

9. Crxnfp similarity of forward and reverse reactions 37

References 38

1. Evaluation metrics and classification models

1.1 Conditional Entropy (CEN)

CEN is an information-theoretic metric that quantifies the remaining uncertainty in the predicted labels given the true labels. It reflects the degree of misclassification across all classes, with lower values indicating higher predictive certainty and better classification performance. Similar to the previous study, the CEN is calculated as follows with confusion matrix (i.e., Matrix):

$$P_{i,j}^{j} = \frac{Matrix(i,j)}{\Sigma_{k=1}^{|C|}\left(Matrix(j,k) + Matrix(k,j)\right)}$$

$$P_{i,j}^{i} = \frac{Matrix(i,j)}{\Sigma_{k=1}^{|C|}\left(Matrix(i,k) + Matrix(k,i)\right)}$$

$$CEN_j = -\Sigma_{k=1,k\neq j}^{|C|}\left(P_{j,k}^{j} log_{2(|C|-1)}\left(P_{j,k}^{j}\right) + P_{k,j}^{j} log_{2(|C|-1)}\left(P_{k,j}^{j}\right)\right)$$

$$P_j = \frac{\Sigma_{k=1}^{|C|}\left(Matrix(j,k) + Matrix(k,j)\right)}{2\Sigma_{k,l=1}^{|C|} Matrix(k,l)}$$

$$CEN = \sum_{j=1}^{|C|} P_j CEN_j$$

1.2 Matthews Correlation Coefficient (MCC)

MCC is a balanced metric that evaluates the quality of binary or multiclass classifications, considering true and false positives and negatives. It returns a value between –1 and +1, where +1 indicates perfect prediction, 0 corresponds to random prediction, and –1 indicates total disagreement between prediction and observation.

$$cov(X,Y) = \sum_{i,j,k=1}^{|C|}\left(\mathrm{Matrix}(i,i)\mathrm{Matrix}(k,j) - \mathrm{Matrix}(j,i)\mathrm{Matrix}(i,k)\right)$$

$$cov(X,X) = \sum_{i=1}^{|C|}\left[\left(\sum_{j=1}^{|C|}\mathrm{Matrix}(j,i)\right)\left(\sum_{k,l=1,k\neq i}^{|C|}\mathrm{Matrix}(l,k)\right)\right]$$

$$cov(Y,Y) = \sum_{i=1}^{|C|}\left[\left(\sum_{j=1}^{|C|}\mathrm{Matrix}(i,j)\right)\left(\sum_{k,l=1,k\neq i}^{|C|}\mathrm{Matrix}(k,l)\right)\right]$$

$$MCC = \frac{cov(X,Y)}{\sqrt{cov(X,X) \times cov(Y,Y)}}$$

1.3 The K-Nearest-Neighbor Classifier

To benchmark with studies using the same dataset, which employed k-nearest neighbor (KNN) models as a baseline, the KNN classifier for the quality evaluation of the proposed reaction representations was implemented using the FAISS framework, developed by Facebook Research.[1] FAISS provides an efficient brute-force k-NN search, enabling its application to relatively large datasets while avoiding potential biases introduced by approximation methods. The number of nearest neighbors was set to k=5 for the test on USPTO 1k TPL dataset and k=3 for the test on EAWAG-BBD/SOIL dataset, since the latter one has a smaller size. The predicted class for a given query was determined by the most frequently occurring class among the retrieved neighbors.

1.4 Multi-Layer Perceptron for Reaction Classification

For the Schneider 50K dataset, the MLP (Multi-Layer Perceptron) was implemented using PyTorch. The model architecture consisted of an input layer with a size corresponding to the input vector, which was 2048 for drfp fingerprints and 256 for rxnfp and crxnfp fingerprints. It was followed by a dense hidden layer with 1664 units and a tanh activation function, and a dense output layer with a softmax activation function. The architecture was consistent with the model design reported in the previous study.[2] For the EAWAG-BBD and EAWAG-SOIL biotransformation reactions, the hidden layer size was reduced to 64, while all other components of the model remained unchanged.

2. Methodology for data visualization

2.1 TMAP

TMAP (Tree-based Mapper)[3] is a scalable and efficient dimensionality reduction and visualization algorithm designed for large-scale and high-dimensional datasets. It constructs a tree-based nearest neighbor graph to represent data in a lower-dimensional space while preserving both local and global structures. In contrast to dimension reduction methods such as t-SNE or UMAP, TMAP leverages cover trees to efficiently approximate neighborhood relationships, allowing it to handle millions of data points with reduced computational cost, and constructs a layout based on locality-sensitive hashing and minimum spanning tree principles, offering a balance between global

overview and local structure retention. The generated plots are displayed using the Matplotlib Python library. TMAP is formally composed of four main algorithmic steps:

Step 1: Locality-Sensitive Hashing (LSH) Forest Construction

Each high dimensional data point $x \in R^d$ is mapped into an LSH forest using MinHash or other LSH variants. This allows for efficient approximate nearest neighbor (ANN) search by reducing the dimensionality via hashing functions $h_1, h_2, \ldots, h_k$ such that $sign({w_i}^T x + b_i)$ for random $w_i \in R^d$, $b_i \in R$.

Step 2: k-Nearest Neighbor Graph Construction

Using the LSH forest, a k-NN graph $G = (V, E)$ is constructed, where each node corresponds to a fingerprint vector and is connected to its k approximate nearest neighbors based on cosine similarity. For vectors x and y, cosine similarity is computed as: $cos(x, y) = \frac{x.y}{|x||y|}$.

Step 3: Minimum Spanning Tree (MST) Extraction

From the k-NN graph, TMAP extracts a minimum spanning tree $T \subseteq G$, ensuring that all nodes are connected in a globally consistent structure with minimized total edge weight. This step preserves the overall topology of the data and avoids cycles.

Step 4: 2D Layout Optimization
Finally, the MST is embedded into a two-dimensional plane using a force-directed layout algorithm that minimizes edge crossings while maintaining neighborhood fidelity. Each node is placed in 2D space such that geometric proximity reflects the similarity of the original high-dimensional fingerprints.

In this study, TMAP was applied to the 256-dimensional crxnfp reaction fingerprints, generated from fine-tuned BERT models. Each fingerprint represents a unique chemical reaction and encodes mechanistically relevant features. Cosine similarity was used as the distance metric for neighbor search in the k-NN graph.

By projecting crxnfp fingerprints into a 2D TMAP layout, we were able to visually inspect the clustering behavior of reactions, assess the preservation of mechanistic similarity, and explore

associations between reaction classes and spatial distribution. Nodes were optionally color-coded by Enzyme Commission (EC) superclass labels or dataset provenance (e.g., Rhea, BBD/SOIL) to facilitate biological interpretation.

2.2 Attention visualization

We extracted attention weights from all 12 transformer layers of the model and averaged these weights across the four attention heads within each layer. In the original BERT model generating rxnfp fingerprints, only the embedding of the [CLS] token was retained. Consequently, for visualization purposes, we focused only on the attention weights corresponding to the [CLS] token for rxnfp fingerprints. In contrast, our crxnfp fingerprints were derived by averaging the embeddings of all tokens rather than relying solely on the [CLS] token. Therefore, we included and averaged the attention weights for all tokens to obtain a comprehensive representation.

The self-attention mechanism in BERT computes the weighted influence of each input token on every other token, defined as:

$$Attention\ (Q, K, V) = softmax(\frac{QK^T}{\sqrt{d_k}})\ V$$

where Q, K, and V represent the query, key, and value matrices derived from the token embeddings, and d_k is the dimensionality of the keys.[4]

We analyzed the multi-head self-attention weights from each of the 12 transformer layers in the BERT models. For rxnfp and rxnfp (NameRxn), attention weights were extracted from the [CLS] token, which is used as a global sequence representation. In the case of crxnfp, where mean-pooled token embeddings are used to represent the reaction fingerprint, we computed attention weights averaged over all non-special tokens in the input SMILES sequence.

Let $\alpha_{l,h,i,j}$ denote the attention weight from token to token i to token j in layer l and head h, where $l \in \{1, \ldots, L\}$, $h \in \{1, \ldots, H\}$, and n is the number of tokens. The average attention weight received by token j at layer l, aggregated over all heads and source tokens, is computed as:

$$\bar{\alpha}_{i,j} = \frac{1}{H_n} \sum_{h=1}^{H} \sum_{i=1}^{n} \alpha_{l,h,i,j}$$

These aggregated weights provide a token-wise attention profile per layer. For crxnfp, this profile was further averaged over all tokens to obtain a single vector summarizing layer-wise attention intensity across the sequence:

$$A_l = \frac{1}{n} \sum_{j=1}^{n} \bar{\alpha}_{l,j}$$

This quantitative analysis of attention weights allows comparison of how information is distributed across tokens and layers under different pooling strategies. The derived metrics form the basis for interpreting token influence and evaluating the behavior of the fingerprinting mechanism across model variants.

3. Distribution of Tanimoto scores before and after transformation

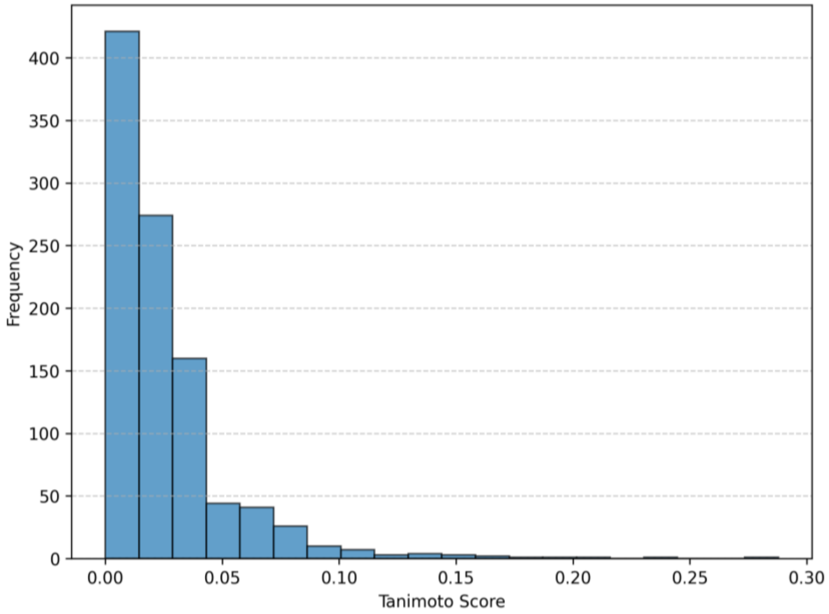

Figure S1. Distribution of Tanimoto scores for randomly selected 1000 reaction pairs.

Figure S1 displays a highly left-skewed distribution of Tanimoto scores from our fine-tuning dataset, with the vast majority of pairwise similarities concentrated near zero. Most scores fall below 0.05, indicating that the sampled reaction pairs are predominantly dissimilar based on the structure-derived drfp fingerprints. Only a small fraction of pairs exhibits moderate similarity (e.g., > 0.1), and values above 0.2 are extremely rare. The skewness highlights the sparsity of structurally similar reaction pairs in the dataset. Given the pronounced left-skew in the distribution of Tanimoto scores, it becomes important to apply a transformation that shifts and stretches the distribution toward higher values. This adjustment is critical for model training, as learning from a highly imbalanced similarity distribution can hinder the model's ability to distinguish between genuinely similar and dissimilar reaction pairs. By transforming the scores—e.g., using a monotonic scaling or a kernel function—we can obtain a more balanced range of similarity values, allowing the model to more effectively learn what constitutes low, moderate, and high similarity.

It is especially important in contrastive or regression-based learning settings, where the model benefits from a more informative and evenly distributed similarity signal.

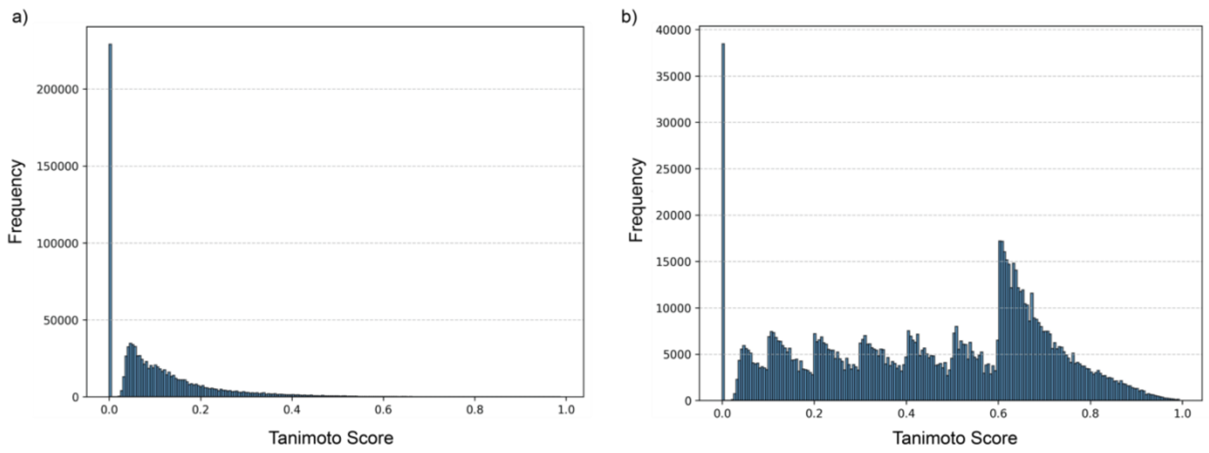

Figure S2. Distribution of transformed Tanimoto scores for a) randomly selected 1 million pairs and b) 1 million target-selected pairs of reactions.

Figure S2b illustrates a more balanced distribution of transformed Tanimoto scores, now spanning the full range from 0 to 1. Unlike the original distribution, which was heavily skewed toward zero, this adjusted distribution ensures that pairwise similarity values are more evenly represented across the spectrum. Within each interval, the local frequency of transformed similarity scores follows the same decreasing trend observed in the overall distribution. However, independently concentrating samples within predefined intervals results in elevated frequencies near the lower boundary of each interval, leading to the discontinuities or saw-tooth patterns observed in Supplementary Figure S2b. The transformation has effectively compressed the overrepresented low-similarity region and stretched underrepresented higher-similarity values, leading to a smoother, denser spread.

This transformation is beneficial for model training, particularly in contrastive learning or similarity regression tasks. By exposing the model to a broad and more uniformly sampled range of similarity scores, the training process can better capture the full gradient of similarity relationships. It allows the model to learn meaningful distinctions not just between extremes (very similar vs. very dissimilar) but also in the intermediate similarity ranges—crucial for nuanced representation learning.

4. Model explainability through extra attention analysis

To investigate the interpretability of the learned reaction fingerprints, we visualized the self-attention weights of BERT-based models across their transformer layers.[5] For models generating rxnfp and rxnfp (NameRxn) fingerprints, we extracted the attention weights corresponding to the [CLS] token,[6] which serves as the global sequence representation. In contrast, for the crxnfp model—which uses mean-pooled token embeddings—we computed the mean attention scores across all tokens for each layer to reflect distributed representational focus.

Attention scores were visualized as two-dimensional heatmaps, with the vertical axis representing transformer layers (from input to output) and the horizontal axis corresponding to SMILES tokens of the input reaction. The intensity of each cell reflects the normalized attention weight, indicating the degree to which a token (atom or bond) is attended to at a specific layer.

These visualizations offer a layer-resolved view of how information flows within the model. They reveal whether the model concentrates attention on reaction-relevant substructures, such as atoms involved in bond rearrangement or functional group transformation. To facilitate interpretation, the reaction schemes were annotated with highlighted reaction centers in both substrates and products, allowing direct comparison between model focus and chemically meaningful features.

rxnfp

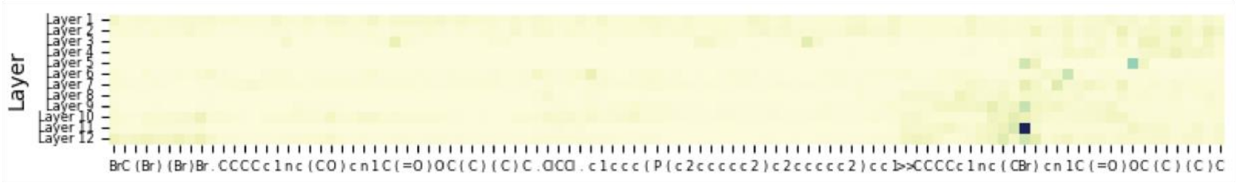

rxnfp (NameRxn)

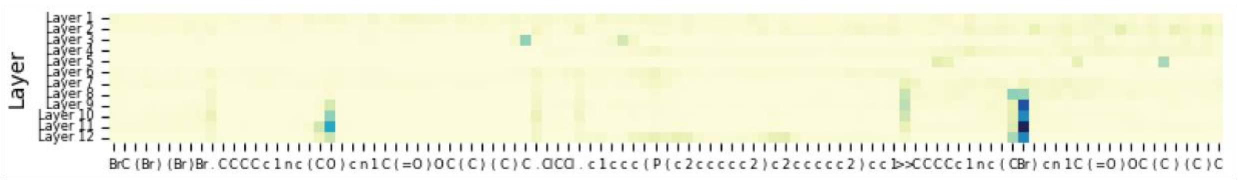

crxnfp

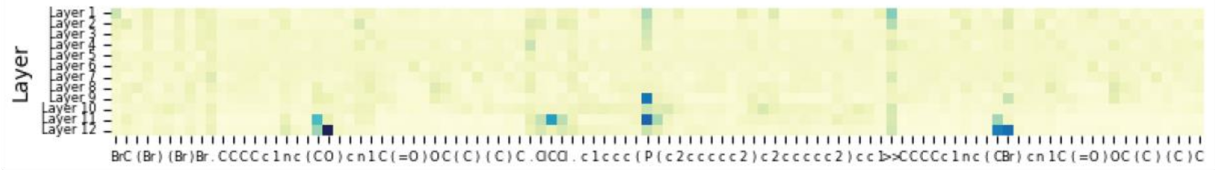

BrC(Br)(Br)Br.CCCCc1nc(CO)cn1C(=O)OC(C)(C)C.ClCCl.c1ccc(P(c2ccccc2)c2ccccc2)cc1>>
CCCCc1nc(CBr)cn1C(=O)OC(C)(C)C

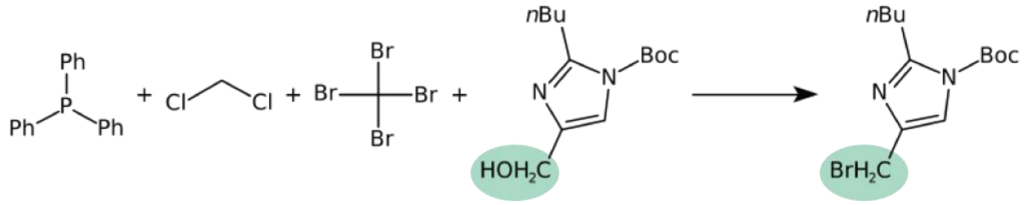

rxnfp

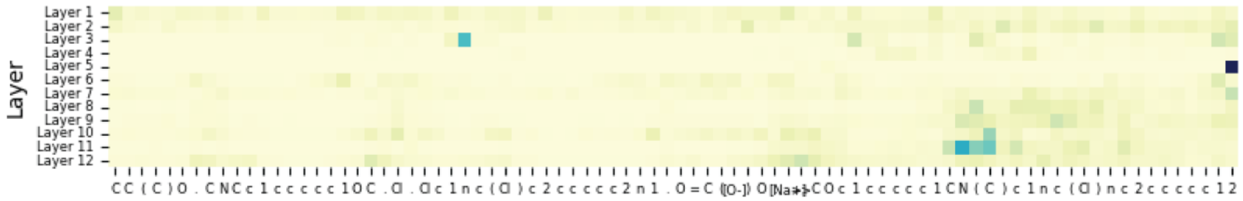

rxnfp (NameRxn)

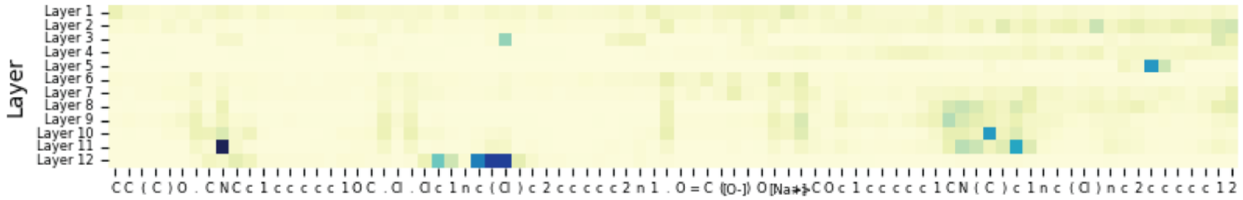

crxnfp

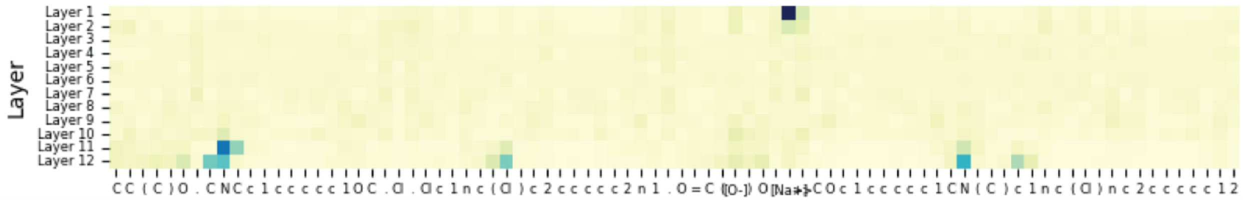

CC(C)O.CNCc1ccccc1OC.Cl.Clc1nc(Cl)c2ccccc2n1.O=C([O-])O.[Na+]>>
COc1ccccc1CN(C)c1nc(Cl)nc2ccccc12

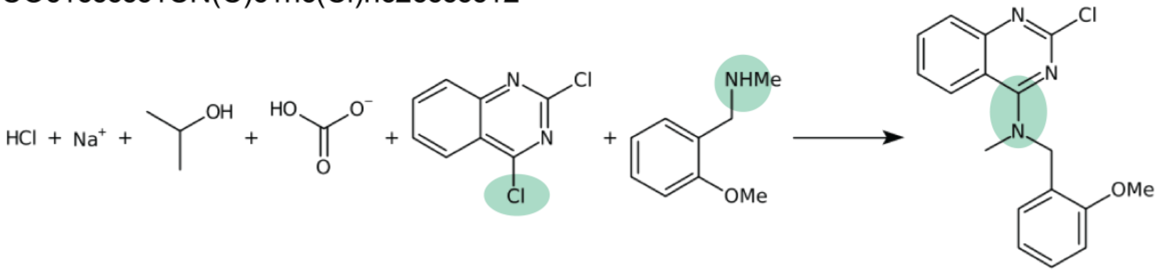

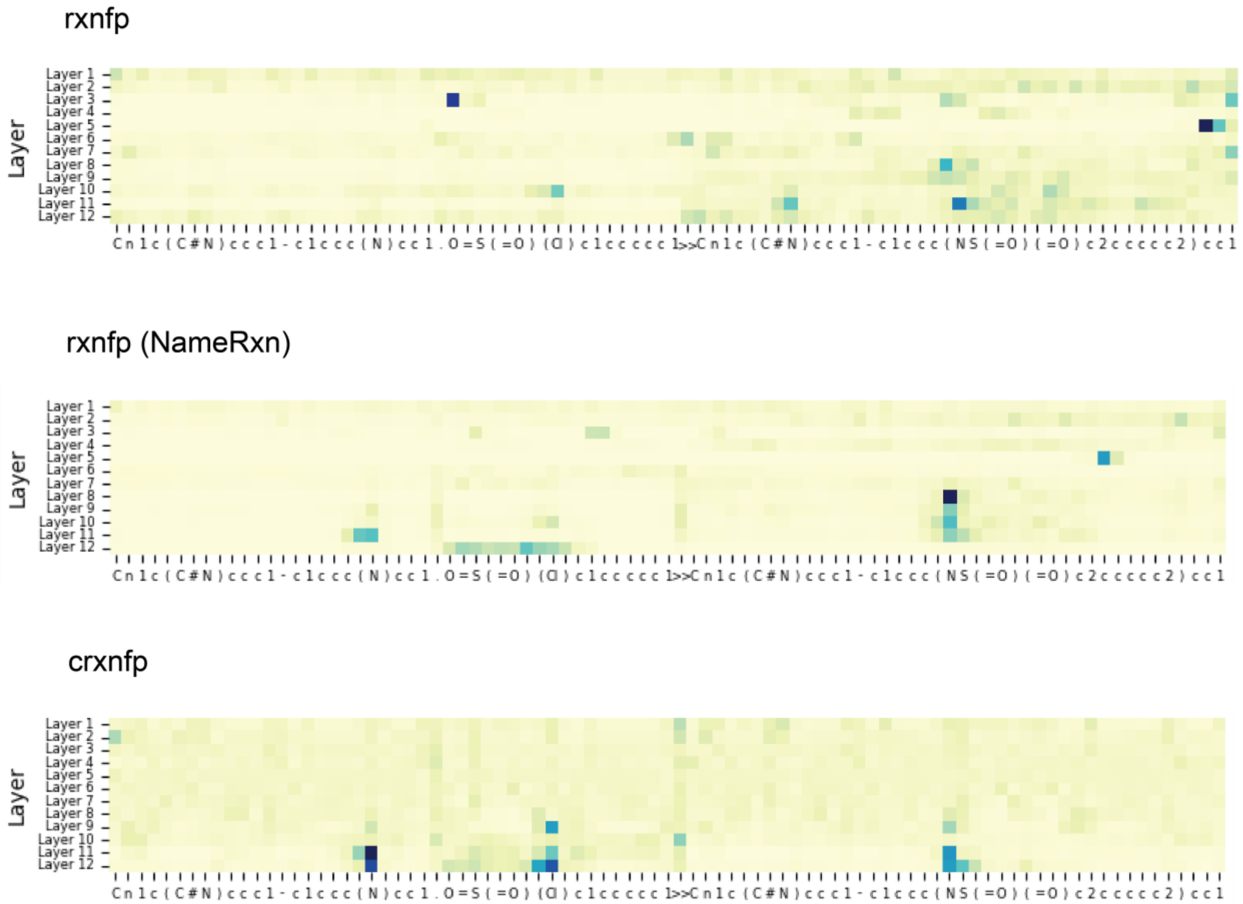

Cn1c(C#N)ccc1-c1ccc(N)cc1.O=S(=O)(Cl)c1ccccc1>>
Cn1c(C#N)ccc1-c1ccc(NS(=O)(=O)c2ccccc2)cc1

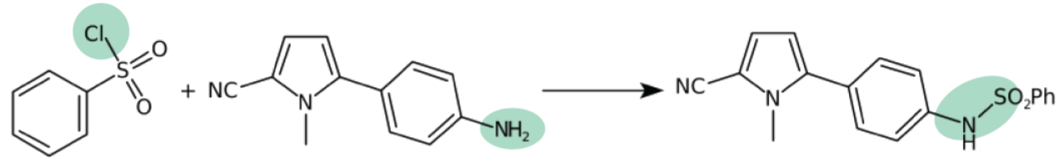

rxnfp

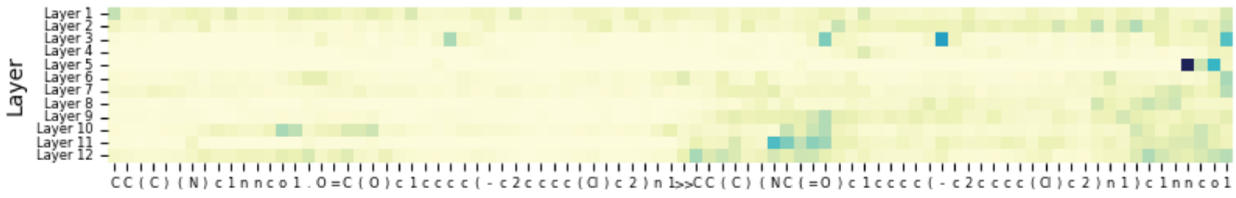

rxnfp (NameRxn)

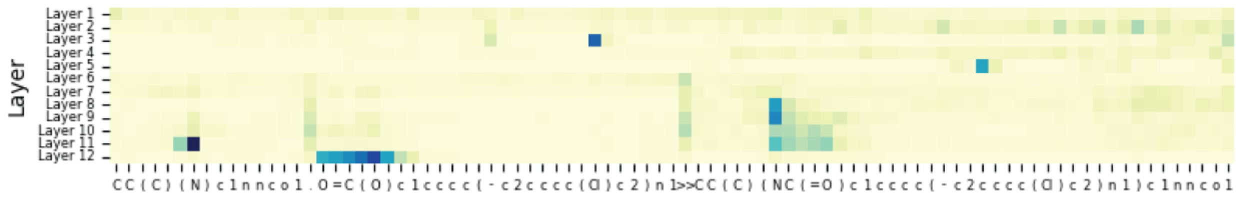

crxnfp

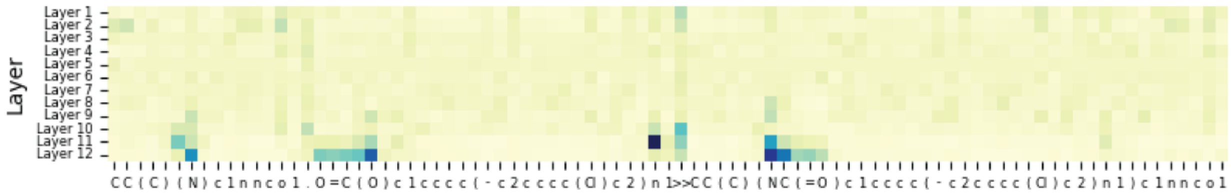

Cn1c(C#N)ccc1-c1ccc(N)cc1.O=S(=O)(Cl)c1ccccc1>>
Cn1c(C#N)ccc1-c1ccc(NS(=O)(=O)c2ccccc2)cc1

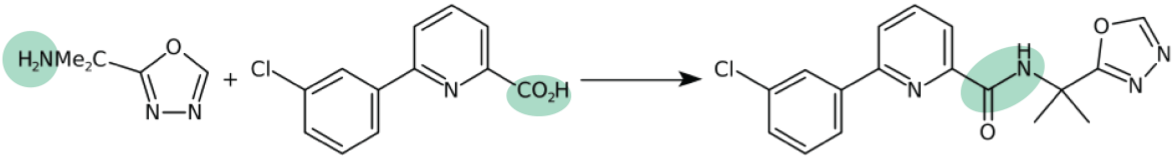

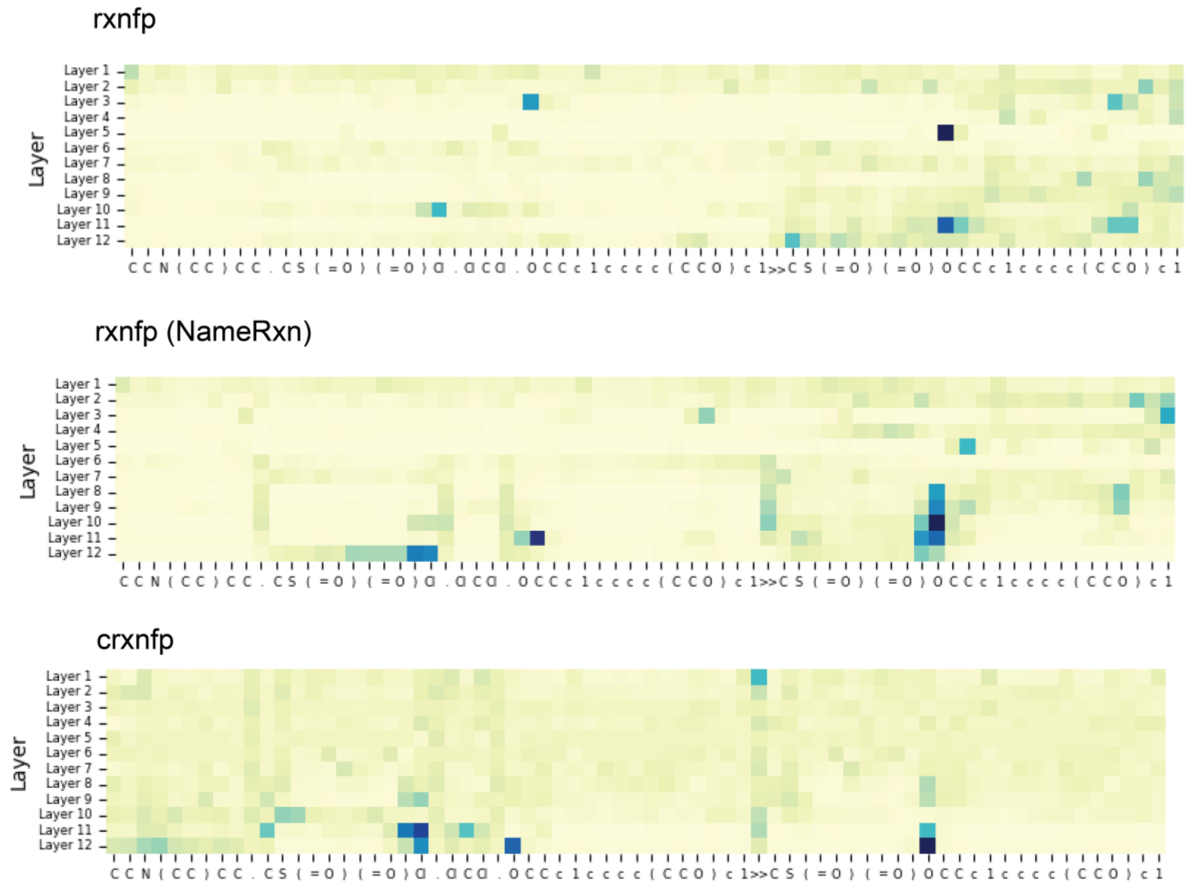

CCN(CC)CC.CS(=O)(=O)Cl.ClCCl.OCCc1cccc(CCO)c1>>
CS(=O)(=O)OCCc1cccc(CCO)c1

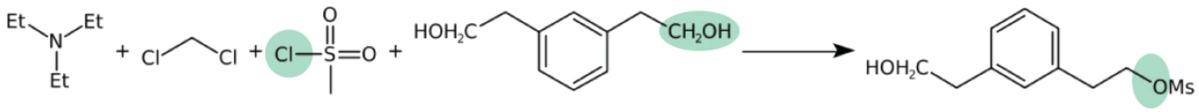

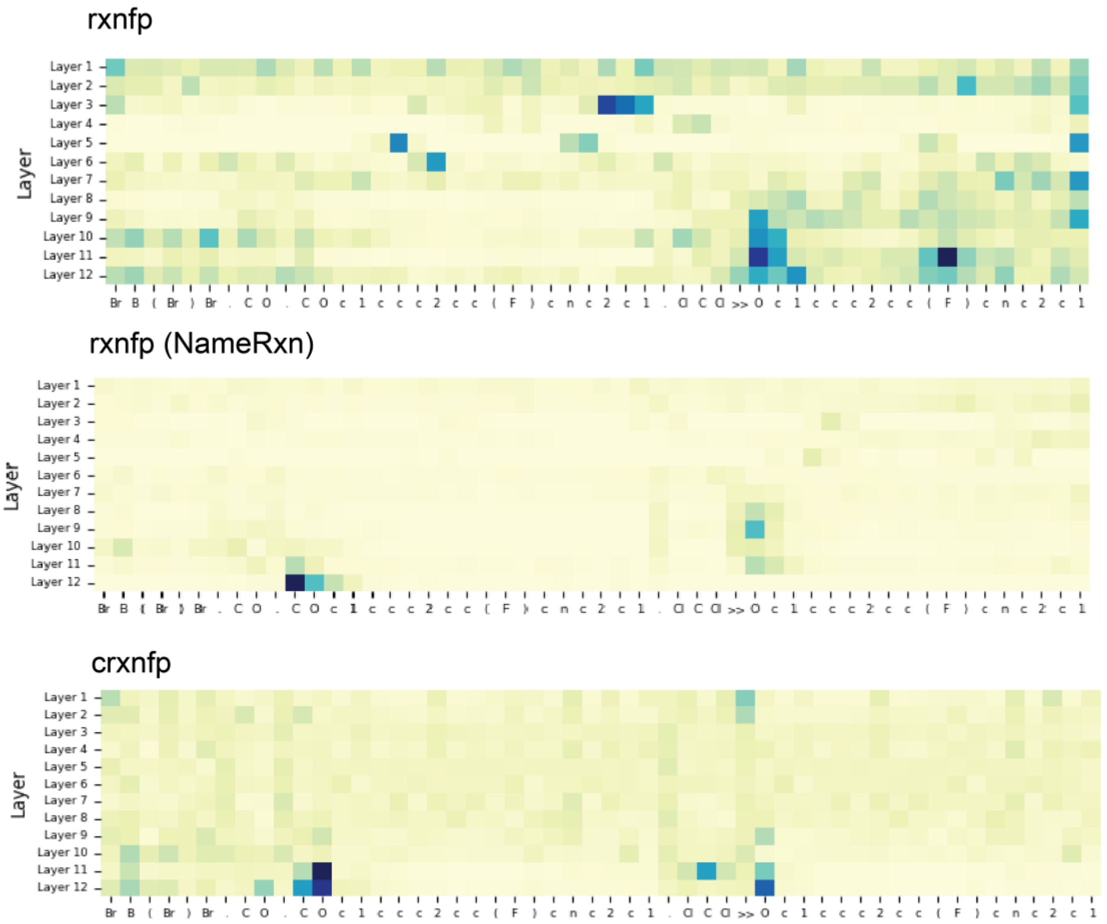

BrB(Br)Br.CO.COc1ccc2cc(F)cnc2c1.ClCCl>>Oc1ccc2cc(F)cnc2c1

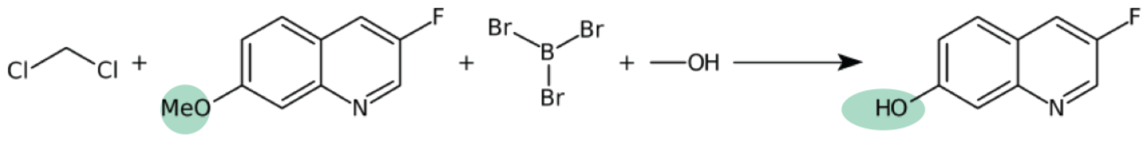

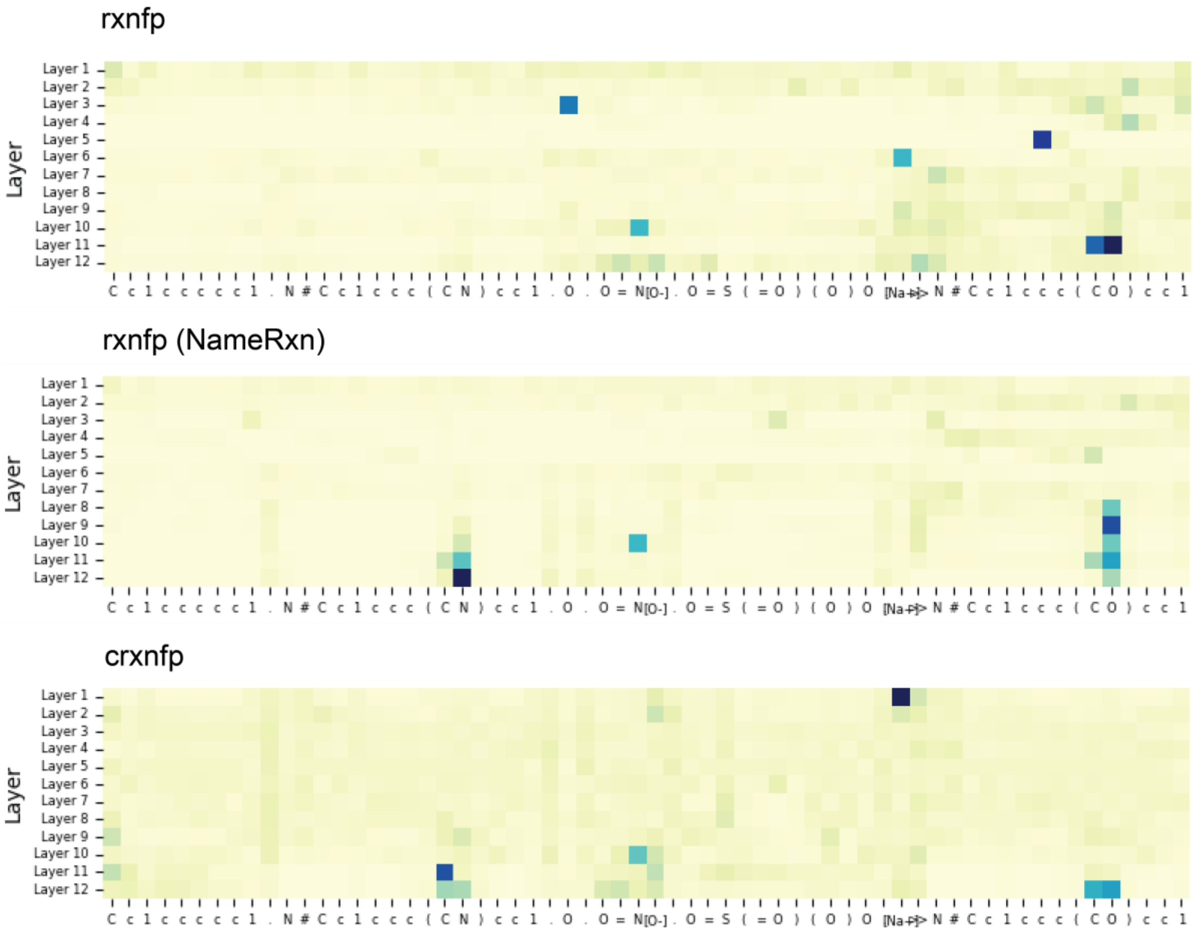

Cc1ccccc1.N#Cc1ccc(CN)cc1.O.O=N[O-].O=S(=O)(O)O.[Na+]>>N#Cc1ccc(CO)cc1

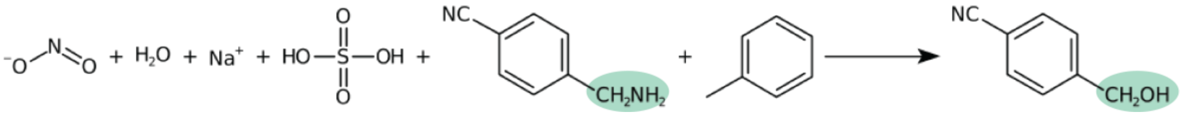

rxnfp

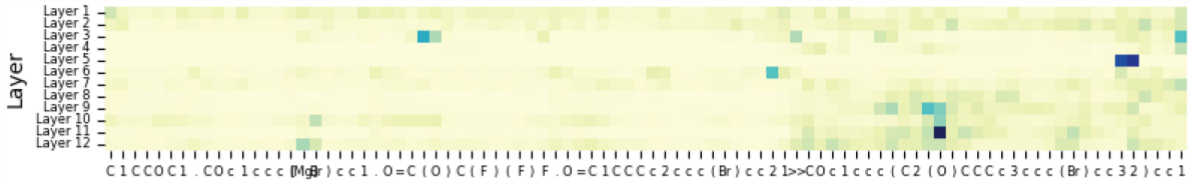

rxnfp (NameRxn)

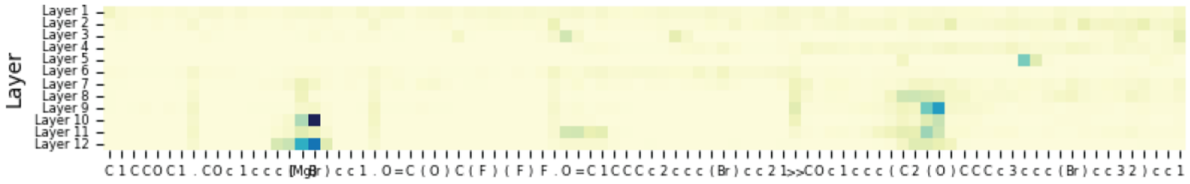

crxnfp

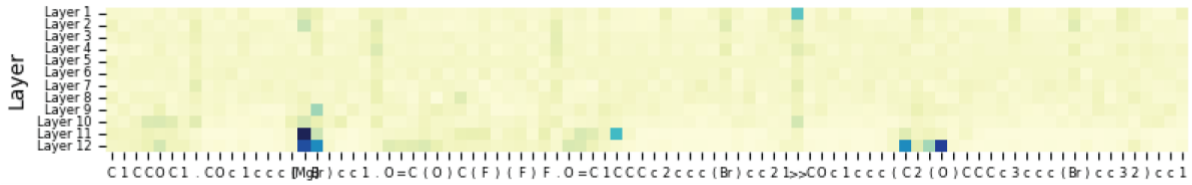

C1CCOC1.COc1ccc([Mg]Br)cc1.O=C(O)C(F)(F)F.O=C1CCCc2ccc(Br)cc21>>
COc1ccc(C2(O)CCCc3ccc(Br)cc32)cc1

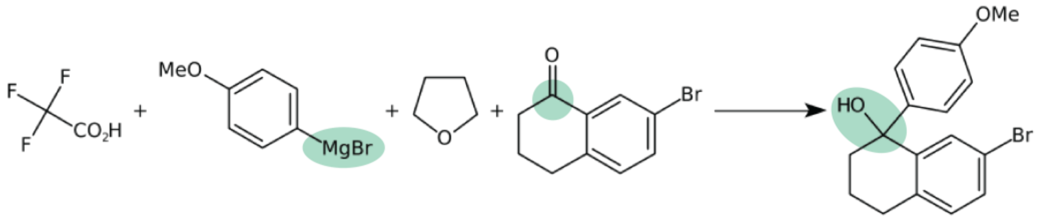

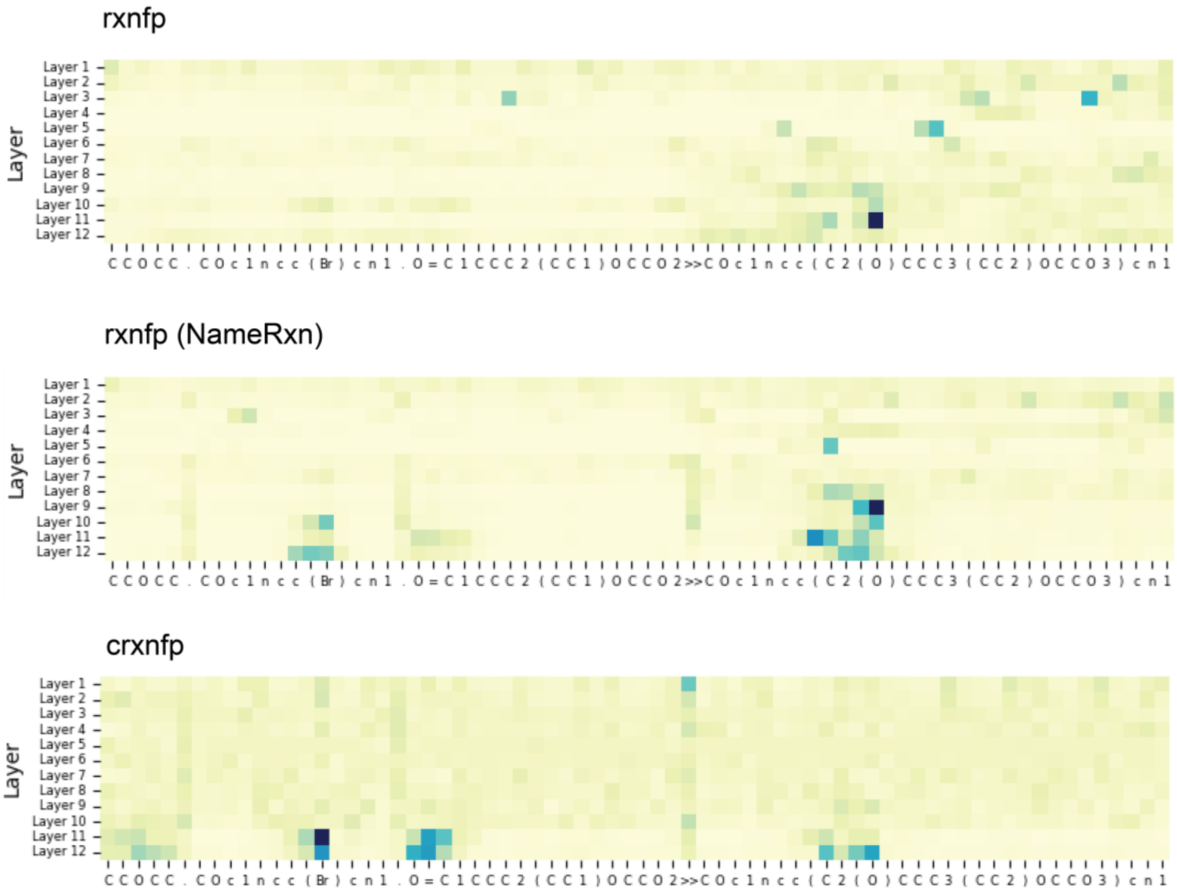

CCOCC.COc1ncc(Br)cn1.O=C1CCC2(CC1)OCCO2>>
COc1ncc(C2(O)CCC3(CC2)OCCO3)cn1

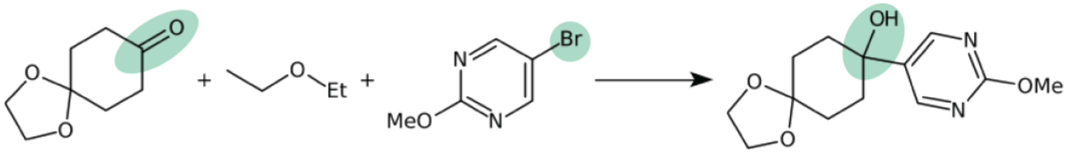

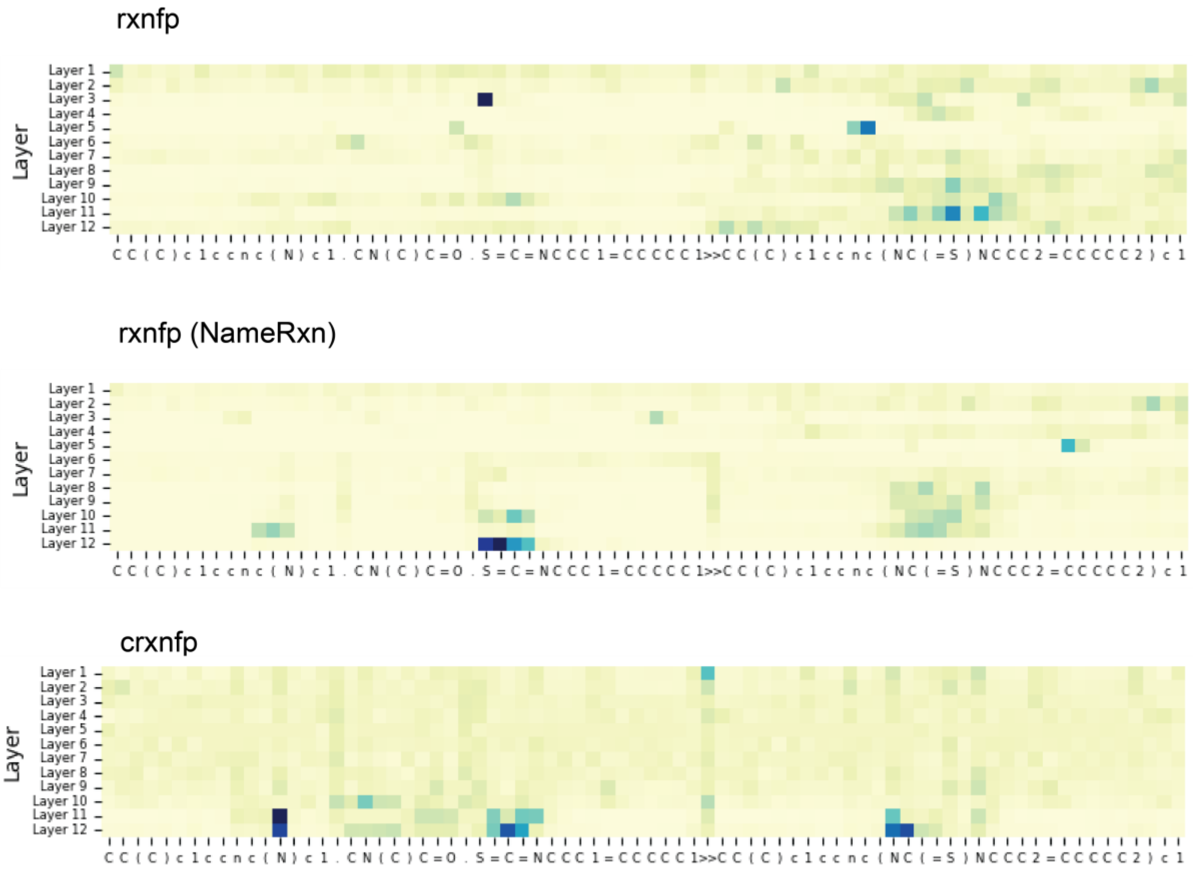

CC(C)c1ccnc(N)c1.CN(C)C=O.S=C=NCCC1=CCCCC1>>
CC(C)c1ccnc(NC(=S)NCCC2=CCCCC2)c1

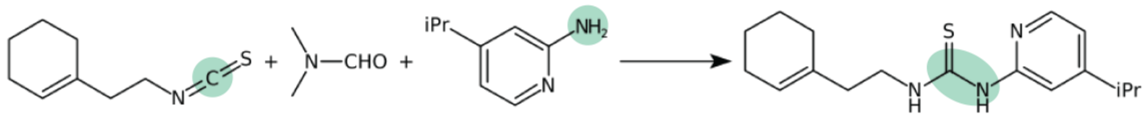

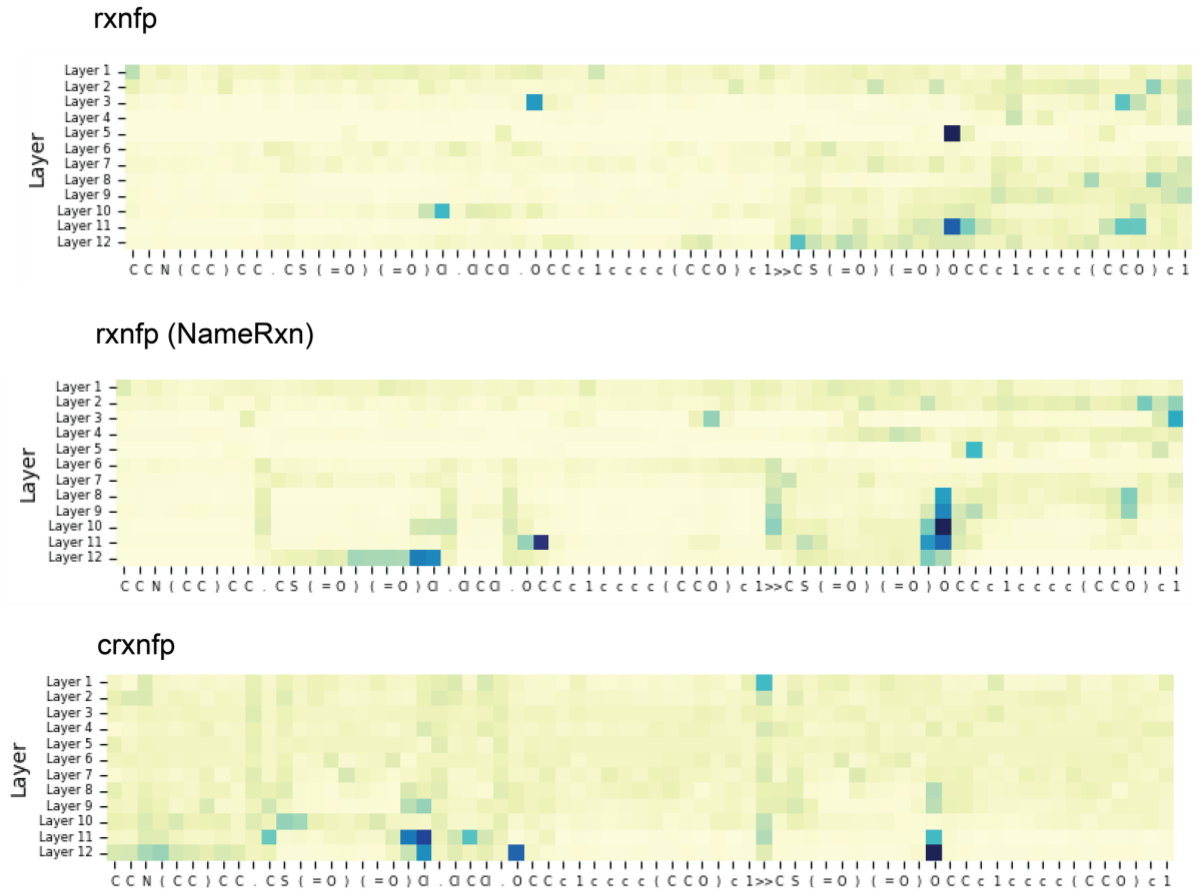

CCN(CC)CC.CS(=O)(=O)Cl.ClCCl.OCCc1cccc(CCO)c1>>
CS(=O)(=O)OCCc1cccc(CCO)c1

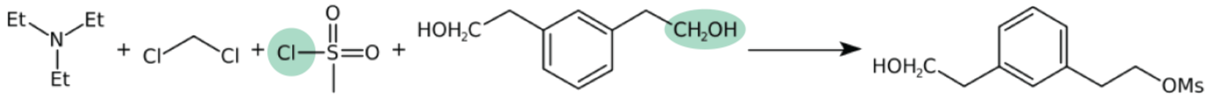

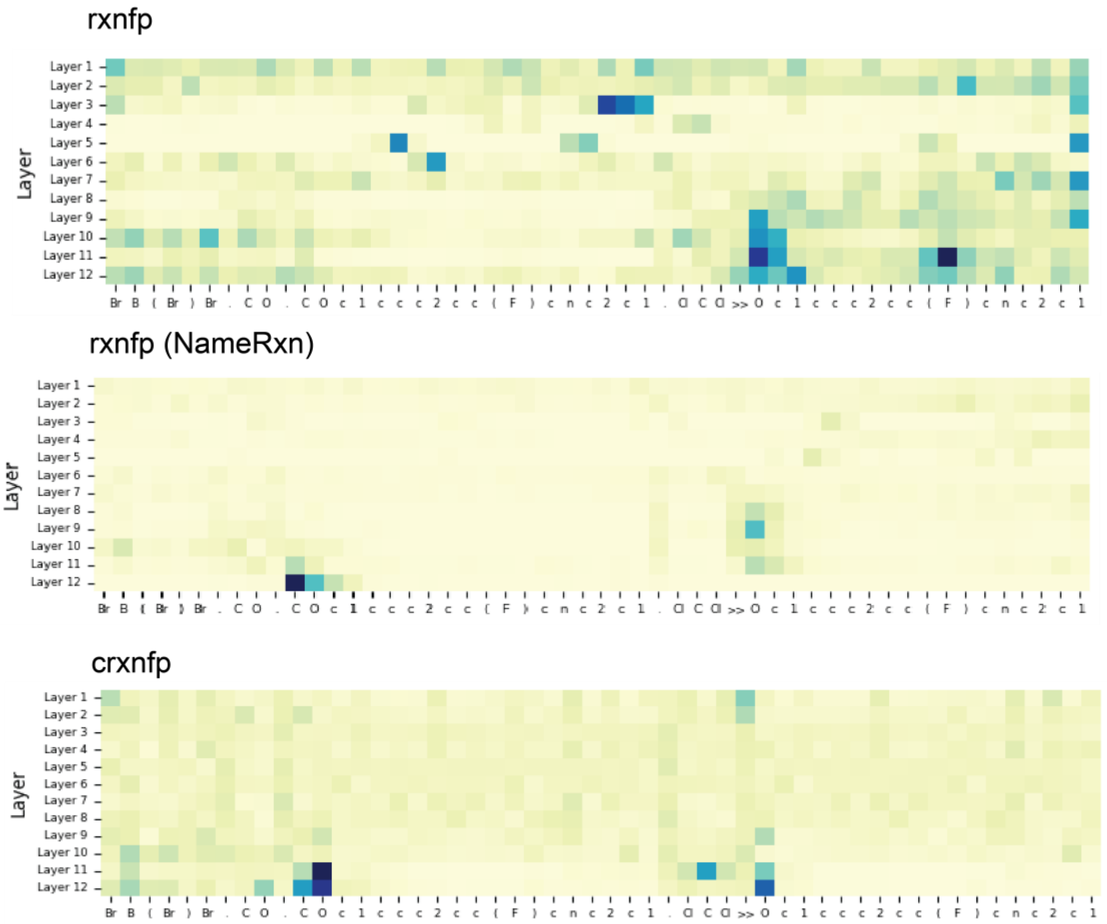

BrB(Br)Br.CO.COc1ccc2cc(F)cnc2c1.ClCCl>>Oc1ccc2cc(F)cnc2c1

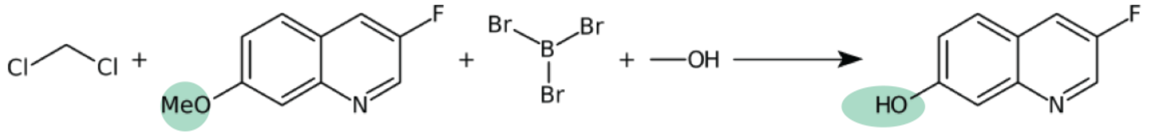

5. Exemplar training reaction pairs with transformed Tanimoto scores

0.86

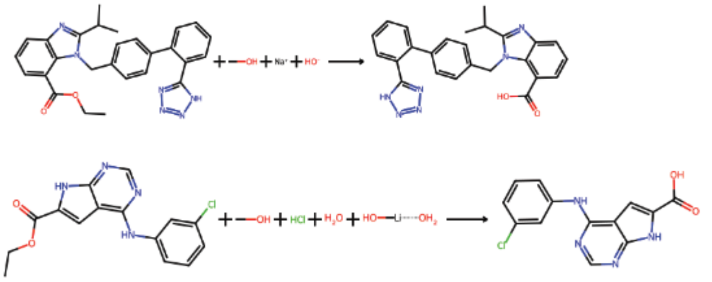

0.05

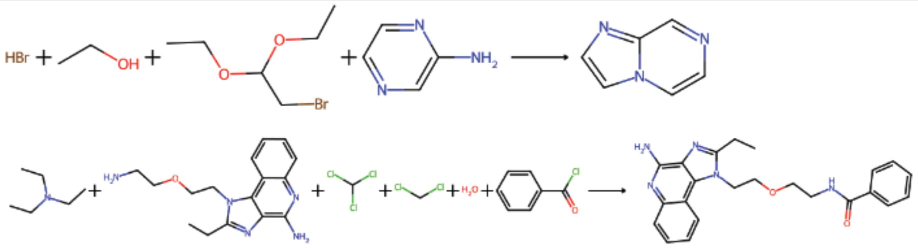

0.58

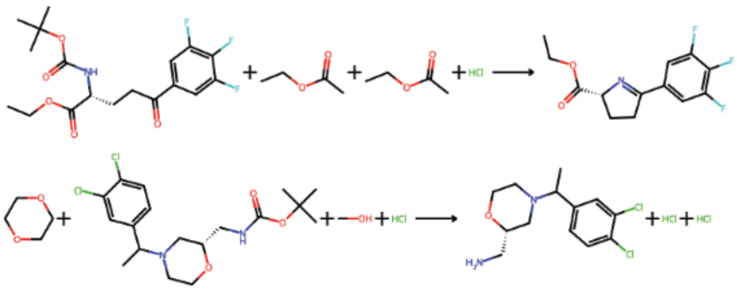

0.65

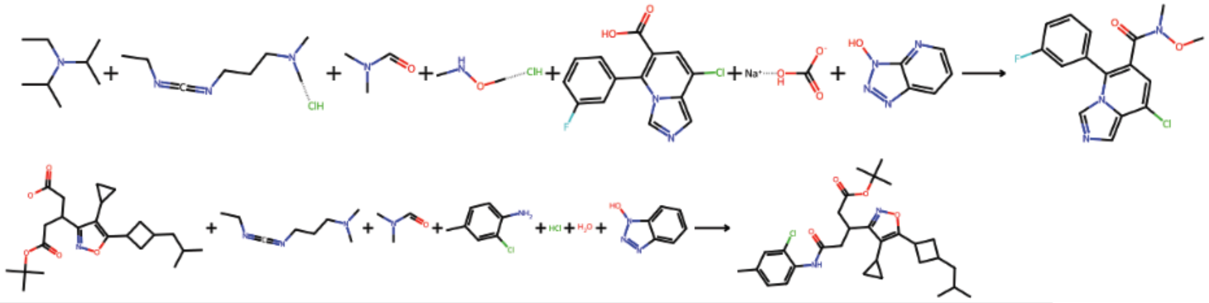

0.14

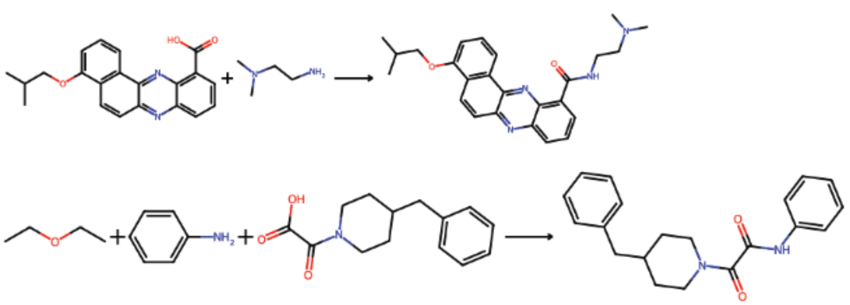

0.31

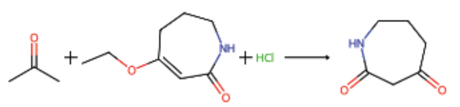

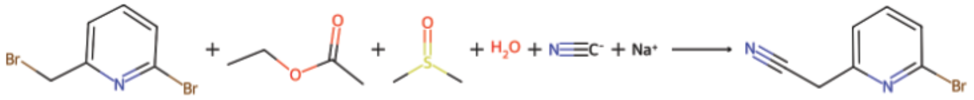

0.36

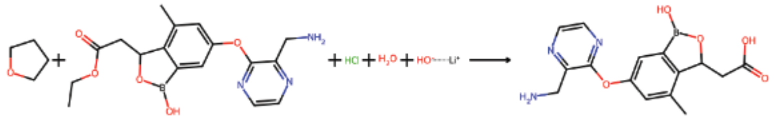

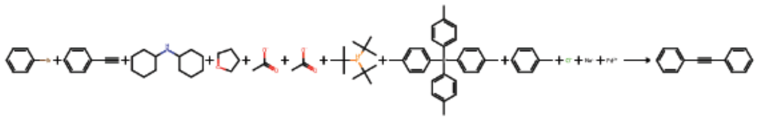

0.83

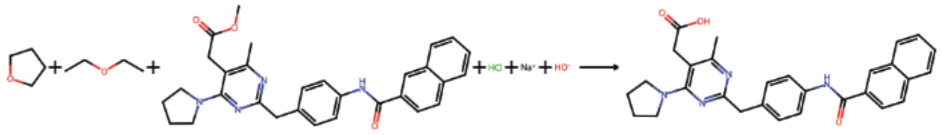

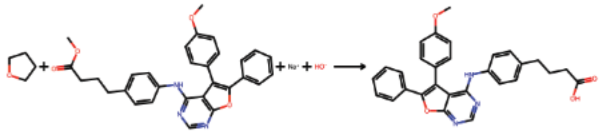

0.63

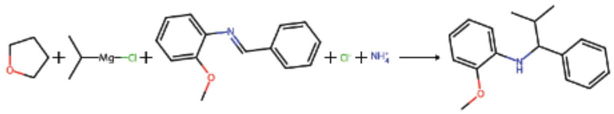

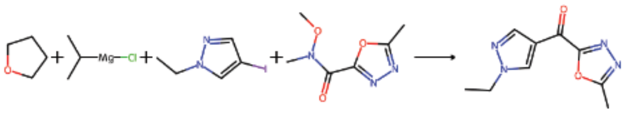

0.75

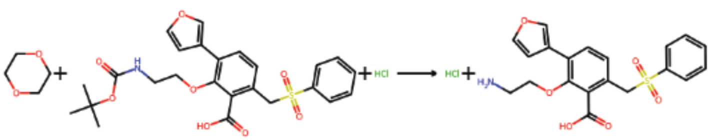

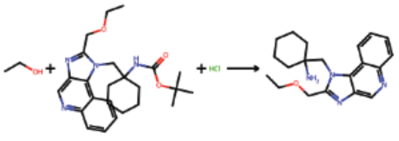

0.56

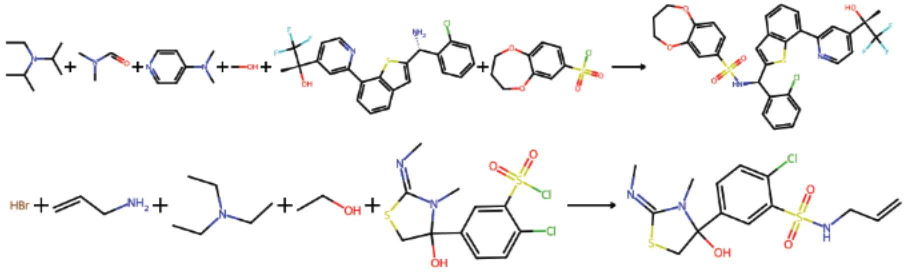

0.36

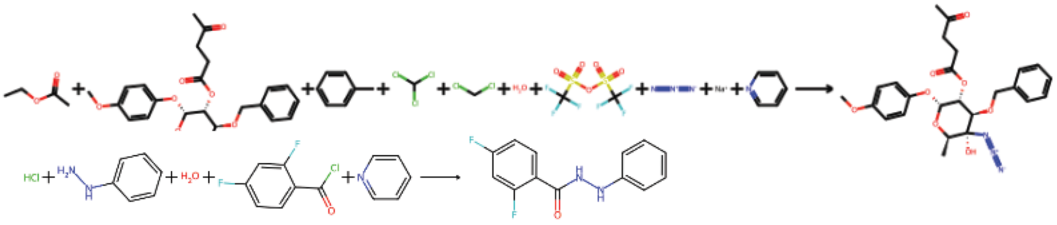

0.66

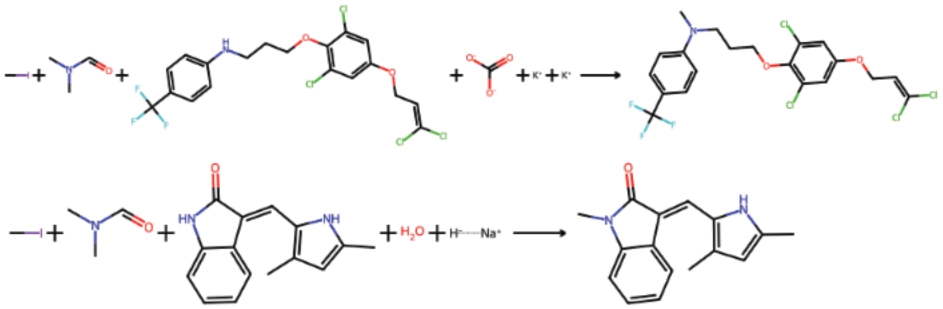

0.10

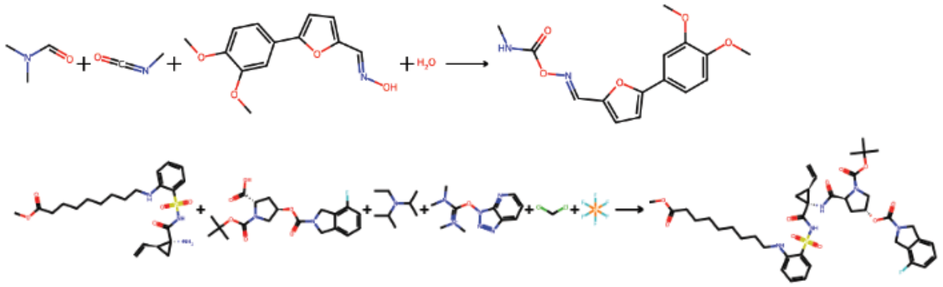

0.52

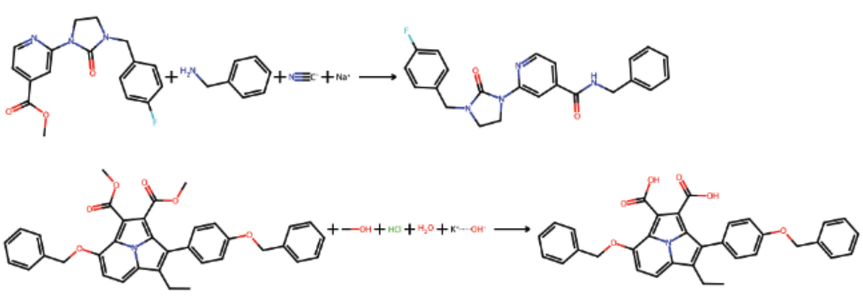

0.70

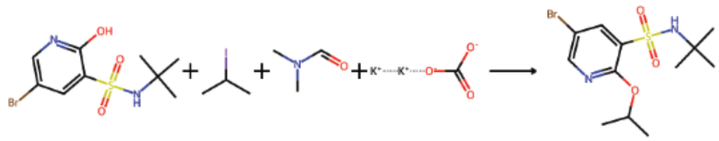

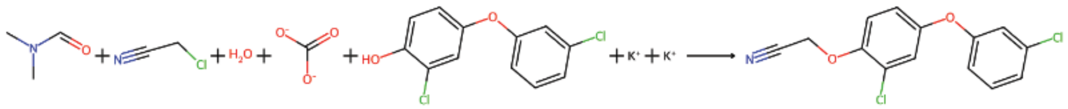

0.43

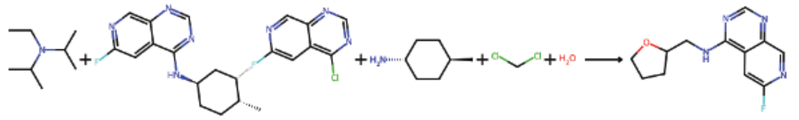

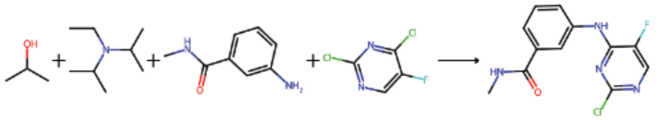

0.64

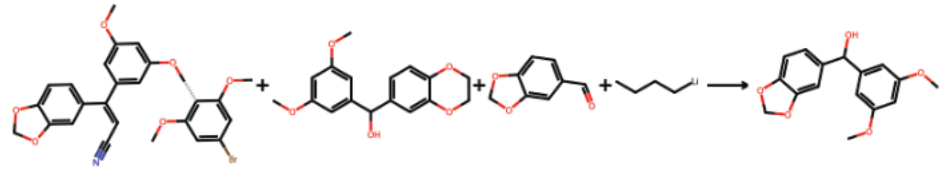

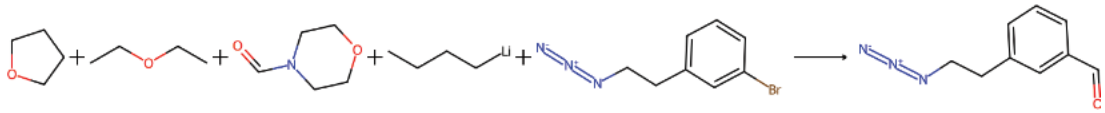

0.37

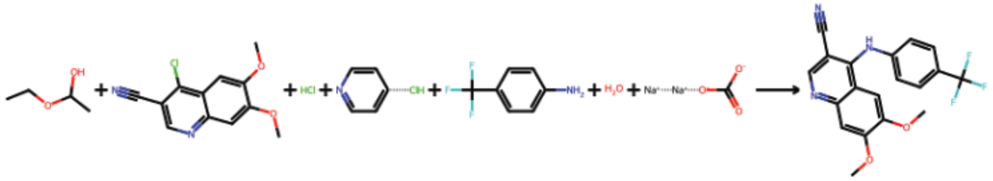

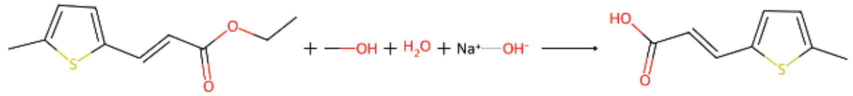

0.64

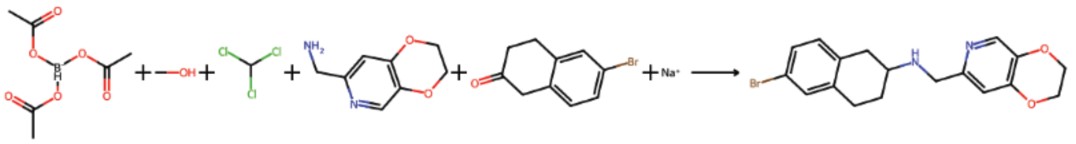

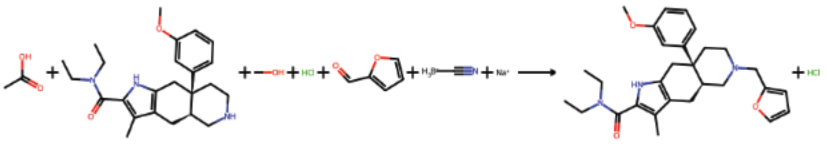

0.00

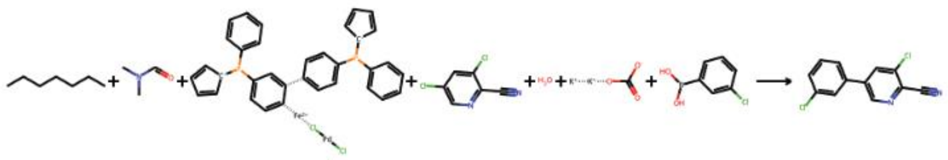

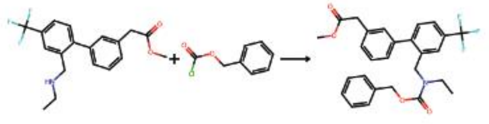

0.69

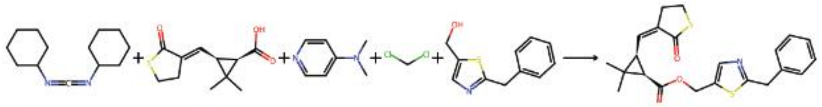

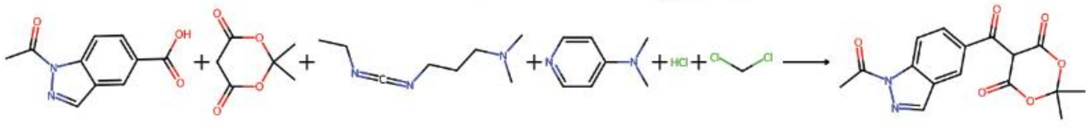

0.62

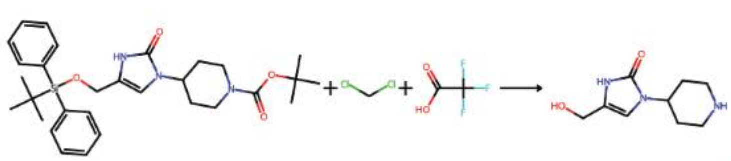

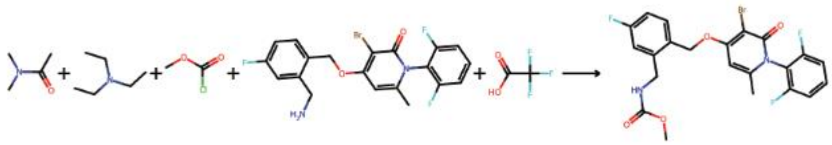

0.00

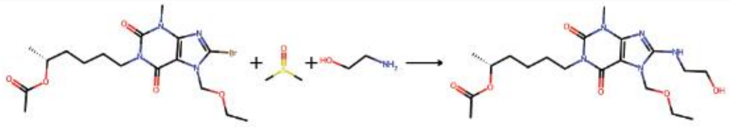

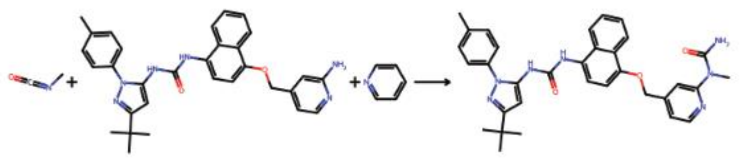

0.70

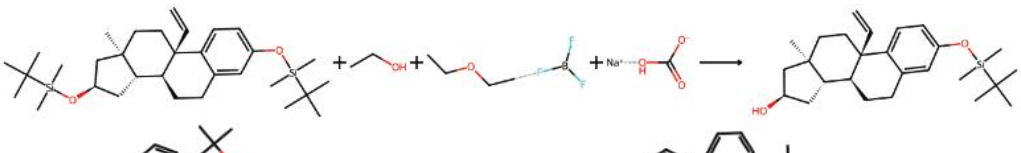

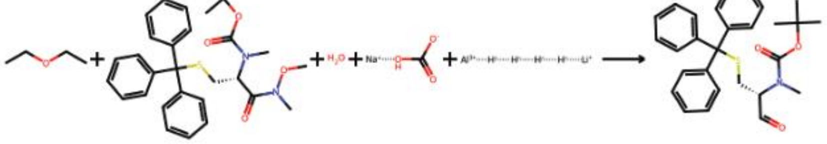

6. Experimental validation of fluorinated compound biotransformation reactions

6.1 Experimental dataset curation

Fluorinated organic compounds, including per- and polyfluoroalkyl substances (PFAS), represent a class of environmentally persistent contaminants characterized by exceptional chemical stability, resistance to microbial degradation, and widespread distribution.[7] In the last few years, microbial defluorination is increasingly recognized as biochemically feasible.[8-10] Although some studies have reported microbial defluorination, the specific enzymes responsible for the transformation remain, in many cases, unidentified.[11, 12] To address this gap, we applied the crxnfp-based enzyme prediction method to a dataset (Table S1) primarily composed of experimentally-validated biotransformation reactions involving fluorinated compounds, along with a small number of curated micropollutant reactions including the hydrolysis of paracetamol and *N,N*-diethyl-3-methylbenzamide (DEET) analogs for the prediction of their EC classes. This dataset includes two enzymes that we experimentally-validated for this study, which catalyze the biotransformation of 5,6-dihydrofluorouracil. These were augmented with literature-derived associations between specific contaminant biotransformations and fourth-level EC classes as a test case for orphan enzyme annotation in an environmental context. The method is based on a similarity-driven strategy, in which crxnfp fingerprints are used to identify annotated reactions with similar transformation patterns. Enzyme annotations are then inferred by transferring EC numbers from similar reactions in the Rhea database to the unannotated query reaction.

Table S1. Visualization of the reactions included in the curated dataset.

Reaction	Reaction Visualization
5-fluorouracil + NADPH → 5,6-dihydrofluorouracil + $NADP^+$	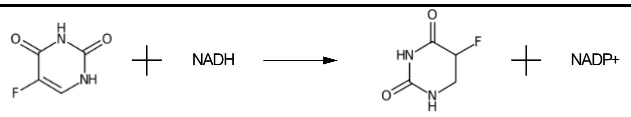
5,6-dihydrofluorouracil + H_2O → 2-fluoro-3-ureidopropionic acid + H^+	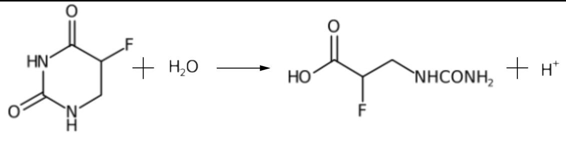

2-fluoro-3-ureidopropionic acid + H_2O + H^+ → 3-amino-2-fluoropropanoic acid + NH_4^+ + CO_2	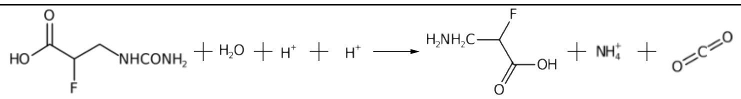
3-amino-2-fluoropropanoic acid + H_2O → 3-aminopropanoic acid + H^+ + F^-	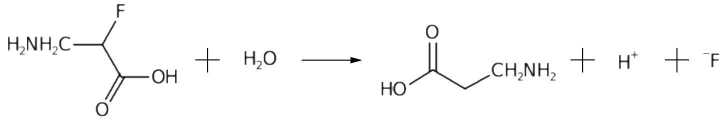
fluoroacetate + H_2O → glycolate + H^+ + F^-	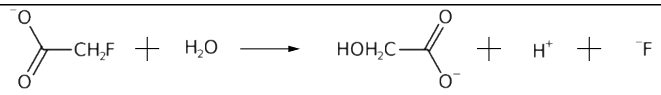
difluoroacetate + H_2O → 2-fluoro-2-hydroxyacetate + H^+ + F^-	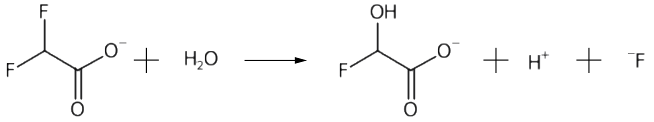
(*E*)-2,3,5,5,5-pentafluoro-4-(trifluoromethyl)pent-2-enoic acid + ATP + CoA → (*E*)-2,3,5,5,5-pentafluoro-4-(trifluoromethyl)pent-2-enoic acid CoA + AMP + diphosphate	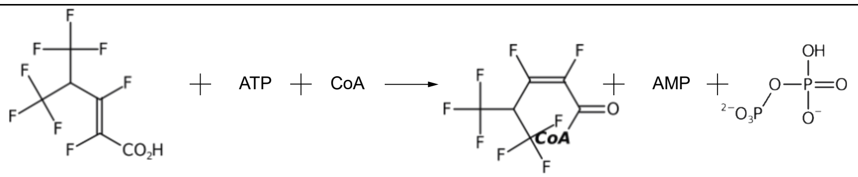
(*E*)-2,3,4,4,5,5,6,6,6-nonafluorohex-2-enoic acid + ATP + CoA → (*E*)-2,3,4,4,5,5,6,6,6-nonafluorohex-2-enoic acid CoA + AMP + diphosphate	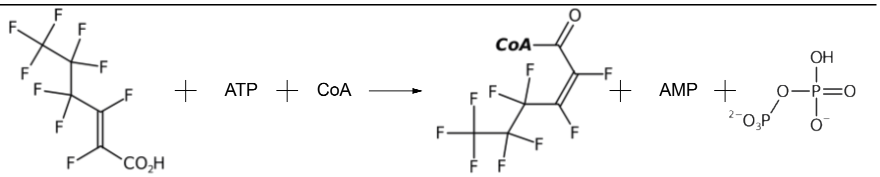
(*E*)-2,3,4,4,5,5,6,6,7,7,8,8,8-tridecafluorooct-2-enoic acid + ATP + CoA → (*E*)-	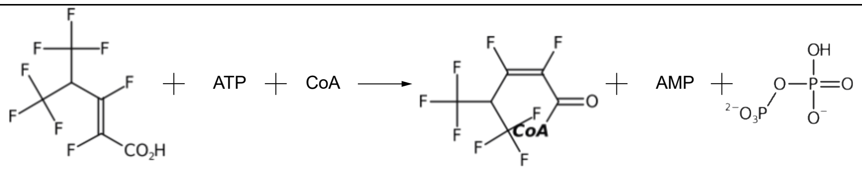

2,3,4,4,5,5,6,6,7,7,8,8,8-tridecafluorooct-2-enoic acid CoA + AMP + diphosphate	
(*E*)-caffeate + ATP + CoA → (*E*)-caffeoyl-CoA + AMP + diphosphate	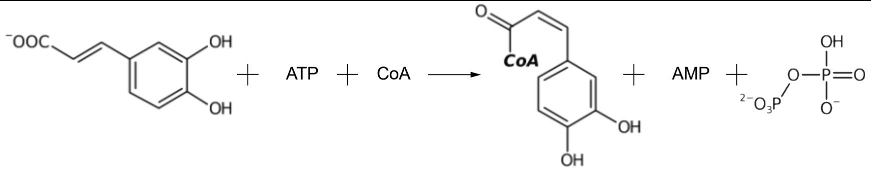
(*E*)-caffeoyl-CoA + oxidized [2Fe-2S]-[ferredoxin] + NADH → hydrocaffeoyl-CoA + reduced [2Fe-2S]-[ferredoxin] + NAD+	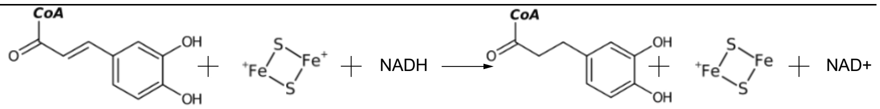
5,5,6,6,6-pentafluorohexanoic acid + ATP + CoA → 5,5,6,6,6-pentafluorohexanoic acid CoA + AMP + diphosphate	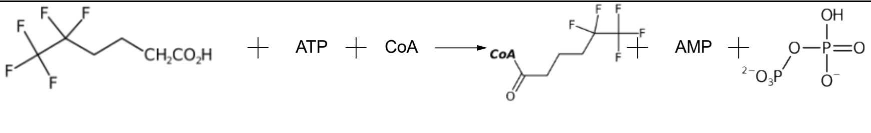
3,3,3-trifluoropropanoic acid + ATP + CoA → 3,3,3-trifluoropropanoic acid CoA + AMP + diphosphate	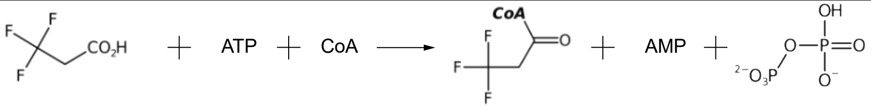
5,5,5-trifluoropentanoic acid + ATP + CoA → 5,5,5-trifluoropentanoic acid CoA + AMP + diphosphate	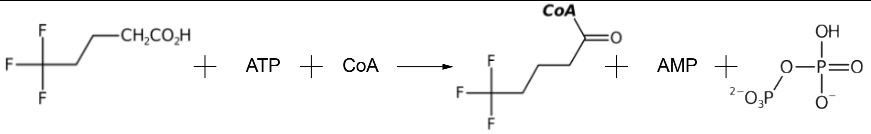
4,5,5-trifluoropent-4-enoic acid + ATP + CoA → 4,5,5-trifluoropent-4-	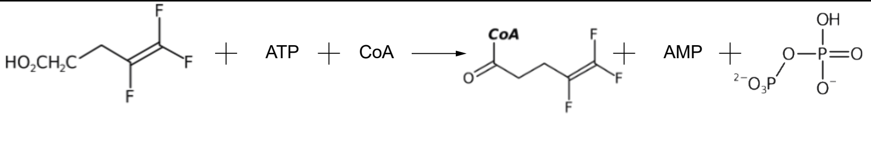

enoic acid CoA + AMP + diphosphate	
4,4,5,5,5-pentafluoropentanoic acid + ATP + CoA → 4,4,5,5,5-pentafluoropentanoic acid CoA + AMP + diphosphate	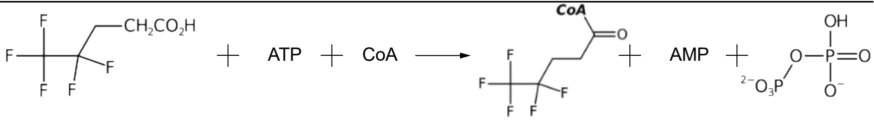
(*E*)-2,3,5,5,5-pentafluoro-4-(trifluoromethyl)pent-2-enoic acid CoA + oxidized [2Fe-2S]-[ferredoxin] + NADH → 2,3,5,5,5-pentafluoro-4-(trifluoromethyl)pentanoic acid CoA + reduced [2Fe-2S]-[ferredoxin] + NAD^+	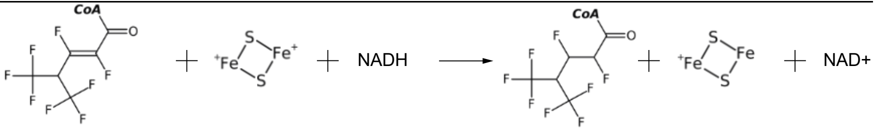
(*E*)-2,3,4,4,5,5,6,6,6-nonafluorohex-2-enoic acid CoA + oxidized [2Fe-2S]-[ferredoxin] + NADH → 2,3,4,4,5,5,6,6,6-nonafluorohexanoic acid CoA + reduced [2Fe-2S]-[ferredoxin] + NAD^+	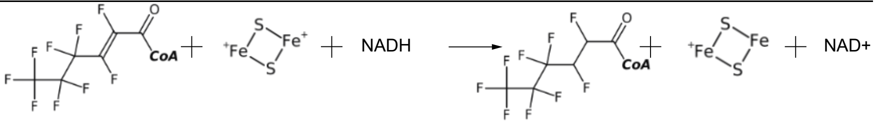
(*E*)-4,6,6,6-tetrafluoro-5-(trifluoromethyl)hex-2-enoic acid CoA + oxidized [2Fe-2S]-[ferredoxin] + NADH → 4,6,6,6-tetrafluoro-5-(trifluoromethyl)hexanoic	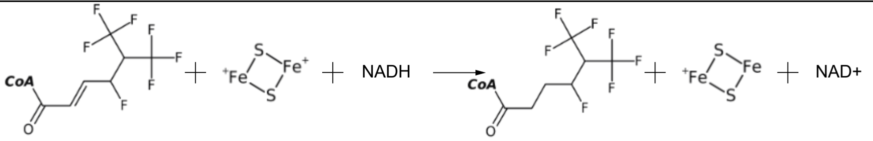

acid CoA + reduced [2Fe-2S]-[ferredoxin] + NAD^+	
(*E*)-2,3,4,4,5,5,6,6,7,7,8,8,8-tridecafluorooct-2-enoic acid CoA + oxidized [2Fe-2S]-[ferredoxin] + NADH → 2,3,4,4,5,5,6,6,7,7,8,8,8-tridecafluorooctanoic acid CoA + reduced [2Fe-2S]-[ferredoxin] + NAD^+	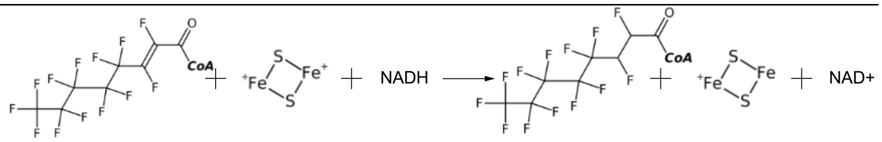
Paracetamol + H_2O → 4-aminophenol + H^+ + acetate	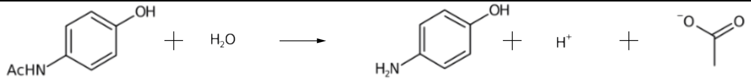
N,N-Diethyl-3-methylbenzamide (DEET) + H_2O → 3-methylbenzoic acid + H^+ + diethylamine	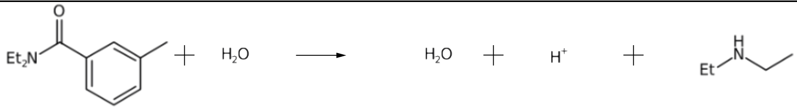
N,N-Dimethyl 4-chlorobenzamide + H_2O → 4-chlorobenzoic acid + H^+ + dimethylamine	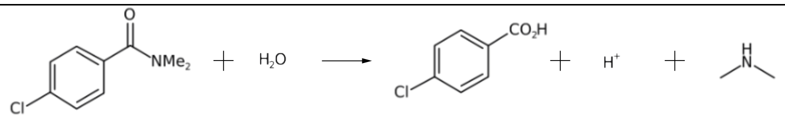

6.2 Cloning, expression and protein purification

Genes encoding enzymes for experimental validation (Table S2) were codon-optimized for *Escherichia coli* using the build-optimization software BOOST[13] and cloned with a C-terminal tobacco etch virus cleavage site and 6x-His tag into pCDFDuet-1 vectors by the Joint Genome Institute. Constructs were expressed in *E. coli* T7 Express cells (NEB C2566I) and cultured in 50 mL of Terrific Broth media supplemented with 50 µg/mL spectinomycin, inoculated from a single colony. After 4 hours of growth, expression was induced with 0.1 mM isopropyl β-d-1-thiogalactopyranoside (IPTG) and incubation continued at 16 °C and 250 rpm for 2 days. The cell cultures (50 mL) were pelleted, resuspended in lysis buffer (20 mM Imidazole, 20 mM Tris-HCl) and sonicated. The lysate was loaded onto a pre-equilibrated column packed with Ni-NTA agarose

beads (Qiagen), subsequently washed twice with wash buffer (40 mM Imidazole, 20 mM Tris-HCl), and eluted in 200 mM Tris-HCl buffer with 500 mM Imidazole. Imidazole was removed from purified protein fractions using a PD-10 column (Cytiva) stored in Salt-Glycerol-Tris buffer (50 mM Tris-HCl, 10% glycerol v/v, 30 mM NaCl,), flash frozen with liquid nitrogen and stored at -80°C. The protein concentration of the eluate was determined using the Qubit™ protein assay kit (Thermo Fisher Scientific).

6.3 Enzyme activity assays

Chemicals for enzyme assays, 5,6-dihydro-5-fluorouracil (substrate) *N*-carbamoyl-2-fluoro-β-alanine (product), were obtained from Chemie Brunschiwg and Santa Cruz Biotechnology, respectively. The purified enzymes WP_095002356.1 and MBS5662960.1 and inactivated enzyme controls were each incubated at a final concentration of 70 µg/mL with 2 mM substrate (5,6-dihydro-5-fluorouracil) in 100 mM Tris-HCl pH 8 at 25°C with total volume of 1 mL. Time points were taken at 0h, 24h and 48h where 150 µL of sample was mixed 1:1 with 150 µL of acetonitrile. Subsequently, the samples were centrifuged at 10,000g for 20 min and the supernatant was analyzed by HPLC. The HPLC method used solvents A (20 mM NaH_2PO_4 + 0.04% H_3PO_4) and B (MeCN + 0.1% 0.04% H_3PO_4) with an Accucore HILIC column (2.6 µm, 150 x 4.6 mm). The flowrate was adjusted to 0.8 mL/min and an isocratic method with 10% A and 90% B for 10 min was used. Compounds were analyzed using HPLC coupled to a UV–vis detection unit (Summit HPLC system, Dionex) with comparison to authentic chemical standards. The decrease in absorbance at 230 nm was monitored and compared to inactivated enzyme controls (Figure S3).

Table S2. Codon-optimized sequences of enzymes newly-characterized in this study. Homology arms compatible with the pCDFDuet-1 vector are shown using a lower case font.

**NCBI Accession**	**Codon-optimized DNA sequence with homology arms (lower case)**
WP_095002356.1	ttttgtttaactttaataaggagatataccATGTCCTTACTTATCCGCGGGGCGAC GCTTGTAACCCACGATGAGTCGTATTGCGCTGATGTTTATTGT GCAGATGGTTTAGTGCGCGCTATTGGAACAGATCTGGATGTT CCACCAGGGTGCGAAGTTGTTGACGGACGCGGCCAGTACTTA ATGCCGGGCGGGATAGATCCTCATACACACATGCAGCTGCCG TTCATGGGCACAGTGGCCAGCGAGGATTTCTTTTCTGGCACA GCGGCCGGTCTGGCAGGGGGAACCACAAGTATAATCGACTT CGTCATTCCCAATCCGCAGCAATCCCTTCTGGAAGCCTTTCAT

	CAGTGGAGGGGATGGGCAGAGAAGTCCGCAGCAGACTACGG ATTCCACGTAGCCATAACTTGGTGGTCCGAACAGGTCAGGGA AGAAATGGCGGAATTAGTGTCCCACCACGGTATCAATAGCTT TAAACACTTTATGGCGTATAAAAACGCGATTATGGCTGCCGA TGATACTCTGGTCGCAAGTTTTGAGCGCTGCCTAGAACTTGG AGCAGTGCCGACAGTGCATGCAGAAAATGGCGAGCTGGTTT ACCACTTACAACGGAAACTGATGGCACAAGGGATGACAGGA CCCGAGGCCCATCCTCTCAGTCGCCCCTCGCAGGTGGAGGGC GAGGCAGCGAGTCGCGCAATTAGGATTGCACAGACGTTGGG TACCCCATTGTATCTTGTGCACGTGAGCACGCGTGAAGCGCT GGATGAGATTACCTACGCTCGTAGCCAAGGCCAGCCCGTTTA TGGCGAAGTTTTAGCGGGGCATCTGTTGTTAGATGATTCGGT TTATCAACACCCCGATTGGCAGACGGCTGCTGGTTATGTTAT GAGCCCACCCTTTCGGCCCCGCGGCCATCAGGAAGCACTCTG GCATGGTTTGCAGAGTGGTAATCTCCATACTACAGCTACAGA TCACTGCTGTTTTTGCGCCGAGCAGAAAGCCGCTGGTCGTGA TGATTTCTCAAAAATTCCAAATGGCACCGCGGGCATAGAGGA CCGGATGGCGCTGTTATGGGATGAGGGGGTGAACACTGGGC GCCTGAGCATGCAGGAGTTTGTGGCCCTGACATCTACCAACA CAGCCAAAATTTTCAACATATATCCTCGCAAAGGCACAATTA GAGTTGGCGCAGACGCAGACCTCGTTTTATGGGATCCTCAAG GGACGAGAACAATCTCAGCTAAAACCCACCACCAAAACGTG GATTTCAATATTTTTGAAGGAAAAACTGTGCGCGGTGTGCCG TCGCACACCATTTCTCAGGGCCGTCTGGTGTGGGTTGACGGA GATTTGCGTGCTGAACGTGGGGCAGGACGCTATATCGAACGC CCCGCCTACCCGGCAGTTTTTGACTTGCTGAAAAAACGGGCT GAACATCAGCAGCCGGTGGCTGTTAAACGCGAGAATCTATAT TTCCAGCATCACCACCATCACCATTAAgatccgaattcgagctcggcgcgc ctgcag
MBS5662960.1	ttttgtttaactttaataaggagatataccATGCGCATCCTTATTAAAAATGGGA CCATCGTTAATGCAAATGGACGGGAACGAAAGGATATATTG ACAGACGGTGATAGAATCATTGCGATCGGAGGTAACCTGGA TGCTGCCGGCGCTGAAGTAATAGATGCAGCAGGGTGCTATGT CATGCCGGGCTTTATCGATACTCATACACACTTCGATCTGGA TGTTGGACTGTGTGTAACTGCGGATAATTTTCGTACCGGCAC GCGCGCGGCGGCACTGGGTGGTACTACCTGTGTGCTAGATTT CGCGACGCAGGATCGCGACGGGACCCTGCGCCAGGCGTTGG AAACCTGGCATAAGAAAGCGGAAGGAAGCTCCTGCAATTAT GGATTTCATATGGCTATAGCGCGTTGGGATGCCGAGACAGAA AAAGAAATGGATTATATGTCTGATAACGGCGTGACTTCCTAT AAAATGTACATGGTATACGACGGCTTAAAGGTCGATGACGG ACAAATATATGCCGCCCTGAAAACCGCACGGGATCACGGCG CTTTAATCGGAATCCACTGCGAAAATTGGGAGGTCCTTCTTC GACGTATAGATGAATTAAAAACCCAGGGGGTCACAGAACCA TGGGGACACCCGATTTCACGCCCGGCGGAGGTCGAAGCCGA

	GGCTGTCGCAAGATATATGCGCATCGCCCAGCTAGCTGGAAC<br>TCCGGCGTATGTTGTCCACCTGTCAACCGAAGAAGGCTTAAT<br>AGAAGCACAGCGTGCTCGTGCCCGTGGCCAAGAAGTCTACCT<br>GGAAACCTGTCCACAGTATCTTTTACTTACAGATGATAGGTA<br>TCGAGACCCGGATGGCGTTAAGTTTATAATGAGTCCGCCGTT<br>GCGCAAGGATGCGGATCGTGAAGCCTTGTGGCAGGGCCTAA<br>AGGAGGGGGCGCTTGACACGATCGGTACTGATCACTGCTCTT<br>TTACTATGGAACAGAAAATGCTCGGTGGGGGCCGCTTCTTTA<br>AAACACCCAACGGAGGTGCCGGAGTTCAACACAGAGGACAG<br>CTGATTTACACTTATGGGGTCTGTGAAGGTCGCCTGACCTTA<br>GAAGATATGGTTAAATATTTGTCTTACAATCCGTCGCGGTTG<br>TTTGGTATGCCAGATAGGGGTGAAATTGCCGAAGAAAAAGC<br>CGCCGATATAGTGATATGGGATCCGTCTGTTAGTGGGACCAT<br>AACAGATACAAATCATGCATATAATTGCGATAATTCAGTATT<br>CGCAGGCTTTGATGTGAAAGGCGCAGCTCGCCACGTGATCAT<br>TAATGGTGAACATATCGTGGAAAACGGTAGCATAAAACTGG<br>AAGGGAGAGGGCGTTATATTTCGCGTACAGGCTATGAGAATC<br>TATATTTCCAGCATCACCACCATCACCATTAAgatccgaattcgagctc<br>ggcgcgcctgcag

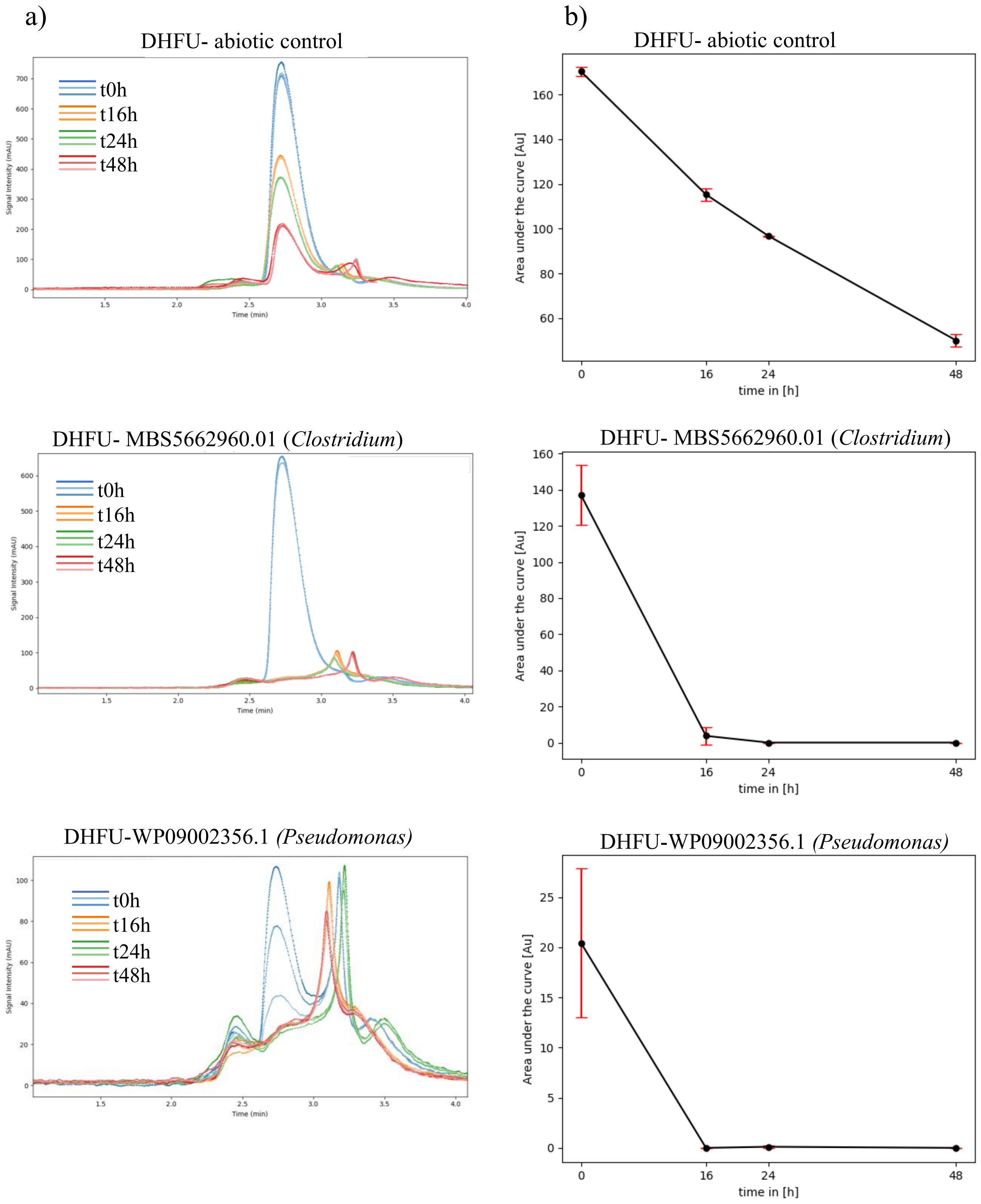
a)
b)
DHFU- abiotic control
DHFU- MBS5662960.01 (*Clostridium*)
DHFU-WP09002356.1 (*Pseudomonas*)
t0h
t16h
t24h
t48h
Signal Intensity (mAU)
Time (min)
Area under the curve [Au]
time in [h]

Figure S3. a) Overlay of HPLC traces of the enzymatic assays with the two chosen PydC proteins. Samples were taken after different timepoints. Blue: 0h; Orange: 16h; Green: 24h and red: 48h. The replicates of the same timepoint are colored in the same color hue. b) Area under the curve at different timepoints.

7. Association of enviPath rules and reactions with UniProt Enzymes

The association of EAWAG-BBD and EAWAG-SOIL biotransformation rules with their corresponding functional enzymes and protein sequences was achieved by retrieving cross-references from the Rhea database to the Swiss-Prot database in UniProt. Swiss-Prot, a high-quality, manually curated subset of the UniProtKB database, contains 572,970 protein sequences.[14] Within these cross-references, a total of 20,393 reactions were associated with UniProt IDs. Among them, 12,717 reactions were undirected, 6,902 reactions proceeded from left to right, and 774 reactions were from right to left. For our analysis, we kept only directional reactions (i.e., left-to-right or right-to-left) and excluded those missing SMILES representations in the Rhea database. After the filtering, 7,575 directed reactions from Rhea that contained both reaction SMILES and associated UniProt IDs were left. Meanwhile, we applied manually curated biotransformation rules from enviPath to EAWAG-BBD and EAWAG-SOIL biotransformation reactions, resulting in 202 rules linked to a total of 1,769 biotransformation reactions. For each of the 1,769 biotransformation reactions in enviPath, we identified the five most similar reactions from the 7,575 Rhea reactions based on the cosine similarity of crxnfp fingerprints. The UniProt IDs associated with the matched Rhea reactions were assigned to the corresponding biotransformation reactions and their linked rules if the cosine similarity exceeded a predefined threshold (i.e., 0.5).

In the first case of Figure 3d), the “reaction_1” from the BBD/SOIL dataset was covered by enviPath rule bt0003, which described the oxidation of an aldehyde to a carboxylic acid. The most similar Rhea reaction identified was labeled as “rhea_reaction_1”, with a cosine similarity of 0.83. According to the Rhea database, this reaction was associated with the UniProt enzyme Q7G9P4, an abscisic-aldehyde oxidase, which was consistent with the transformation type description in bt0003 provided by experts. In the second example, we analyzed enviPath rule bt0361 and identified the most similar Rhea reaction corresponding to the associated biotransformation. This Rhea reaction was linked to multiple UniProt enzymes, all annotated as Autotaxin, which aligned well with the known mechanisms of the biotransformation.

8. Fine-tuning progress of large language models

We compared the learning dynamics during fine-tuning of two approaches for generating crxnfp fingerprints, one using the [CLS] token embedding and the other relying on mean-pooled token embeddings. The underlying model configuration, training conditions, and optimization procedure were kept consistent between experiments.

The evaluation loss (i.e., MSE) over the course of training can be found in Figure S4. The model utilizing average pooling over token embeddings showed both faster convergence and a lower final validation error, achieving a minimum MSE of 0.0037. Additionally, it achieved a Pearson correlation of 0.97 between the cosine similarity of crxnfp fingerprints and the Tanimoto scores of drfp fingerprints. In contrast, the model relying solely on the [CLS] token representation converged more slowly and reached a higher loss of 0.0057, alongside a slightly lower Pearson correlation of 0.96 between the cosine similarity of crxnfp fingerprints and the Tanimoto scores of drfp fingerprints. These results suggested that the averaged embeddings provided a slightly more robust and generalizable representation of chemical reactions in our case. Throughout training, the mean-pooled model maintained a smoother trajectory, with less fluctuation in validation loss. The only observed drop in validation loss around 40,000 steps may be attributed to the progressively decreasing learning rate during training. This stability indicated a more consistent learning signal, potentially due to the more comprehensive information captured when aggregating across all token embeddings. Meanwhile, the [CLS]-based model had higher variance between evaluation checkpoints, suggesting greater sensitivity to specific training samples or noise. Considering both the improved validation loss and the smoother convergence behavior observed for the mean-pooled embedding model, these results evidenced a potential benefit of leveraging distributed token-level information rather than relying solely on a single-position embedding. In the context of reaction similarity search, pooling strategies that incorporate broader contextual information may offer improvements in both predictive performance and training consistency. Therefore, for all the analysis in this work, we applied mean-pooled token embeddings as the crxnfp fingerprints.

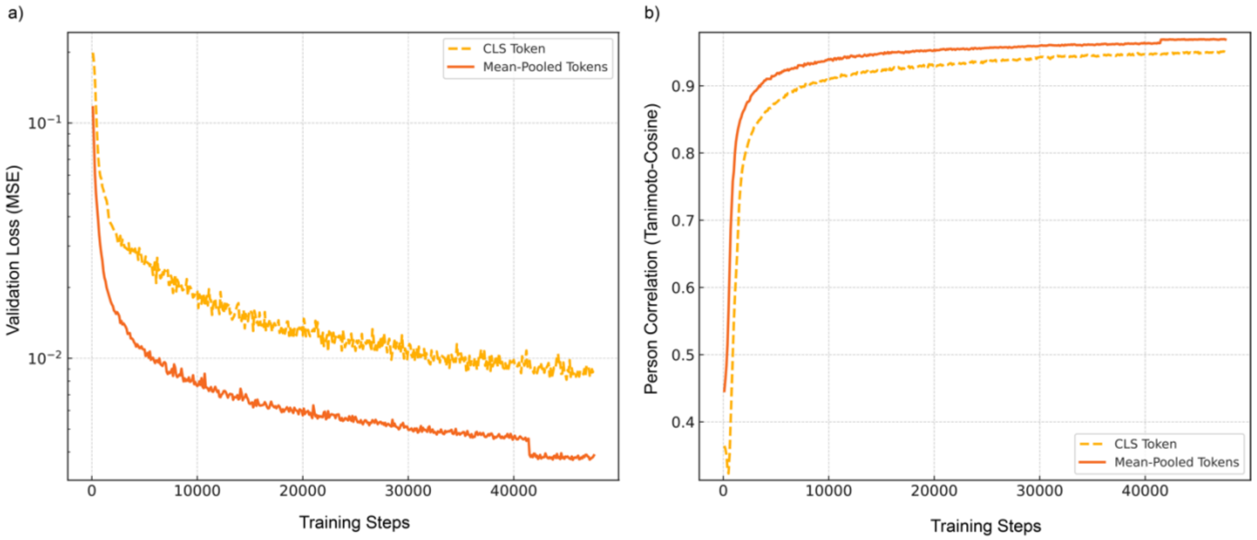

Figure S4. Fine-tuning performance comparison of strategies for generating crxnfp fingerprints. a) Validation loss (i.e., MSE) over training steps. b) Pearson correlation between the cosine similarity of crxnfp fingerprints and the Tanimoto similarity of drfp fingerprints. Two fingerprinting strategies were compared using the embedding of the [CLS] token and the mean-pooled embeddings across all tokens.

9. Crxnfp similarity of forward and reverse reactions

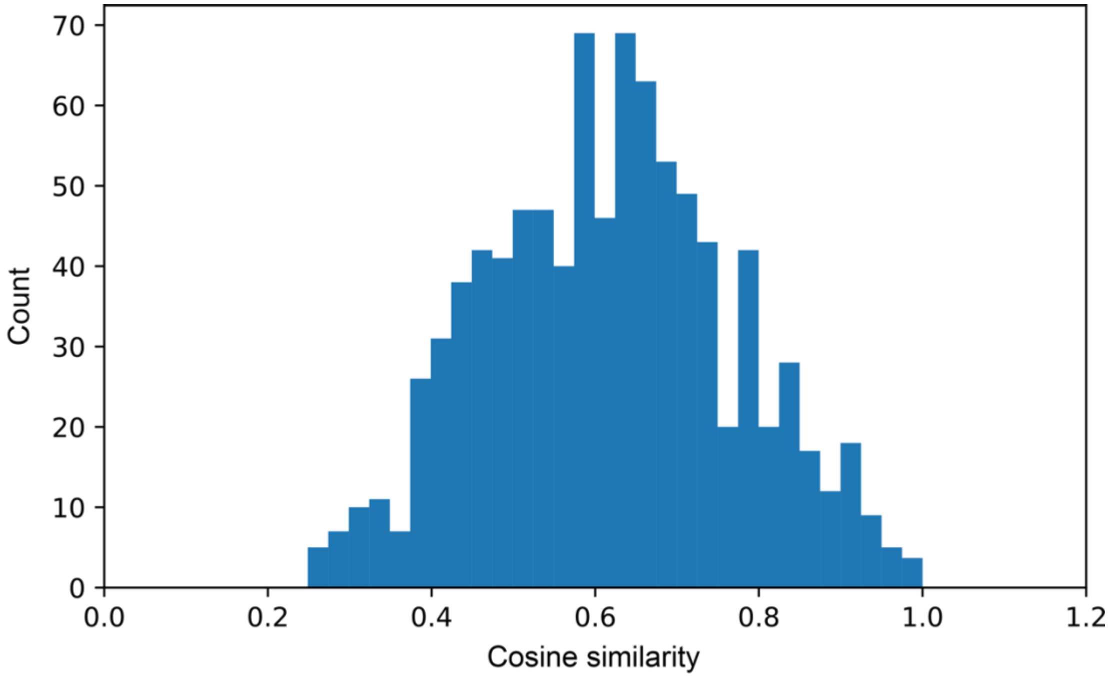

Figure S5. Distribution of cosine similarities between crxnfp fingerprints computed for randomly selected 1,000 pairs of forward and reverse Rhea reactions, exploring the influence of reaction directionality on the learned embedding space.

References

1. Johnson, J.; Douze, M.; Jégou, H., Billion-scale similarity search with GPUs. *IEEE Transactions on Big Data* **2019,** *7* (3), 535-547.
2. Probst, D.; Schwaller, P.; Reymond, J.-L., Reaction classification and yield prediction using the differential reaction fingerprint DRFP. *Digital discovery* **2022,** *1* (2), 91-97.
3. Probst, D.; Reymond, J.-L., Visualization of very large high-dimensional data sets as minimum spanning trees. *Journal of Cheminformatics* **2020,** *12* (1), 12.
4. Vaswani, A.; Shazeer, N.; Parmar, N.; Uszkoreit, J.; Jones, L.; Gomez, A. N.; Kaiser, Ł.; Polosukhin, I., Attention is all you need. *Advances in neural information processing systems* **2017,** *30*.
5. Devlin, J.; Chang, M.-W.; Lee, K.; Toutanova, K. In *Bert: Pre-training of deep bidirectional transformers for language understanding*, Proceedings of the 2019 conference of the North American chapter of the association for computational linguistics: human language technologies, volume 1 (long and short papers), 2019; pp 4171-4186.
6. Schwaller, P.; Probst, D.; Vaucher, A. C.; Nair, V. H.; Kreutter, D.; Laino, T.; Reymond, J.-L., Mapping the space of chemical reactions using attention-based neural networks. *Nature machine intelligence* **2021,** *3* (2), 144-152.
7. Wackett, L. P.; Robinson, S. L., A prescription for engineering PFAS biodegradation. *Biochemical Journal* **2024,** *481* (23), 1757-1770.
8. Marciesky, M.; Aga, D. S.; Bradley, I. M.; Aich, N.; Ng, C., Mechanisms and opportunities for rational in silico design of enzymes to degrade per-and polyfluoroalkyl substances (PFAS). *Journal of Chemical Information and Modeling* **2023,** *63* (23), 7299-7319.
9. Hu, M.; Scott, C., Toward the development of a molecular toolkit for the microbial remediation of per-and polyfluoroalkyl substances. *Applied and Environmental Microbiology* **2024,** *90* (4), e00157-24.
10. Probst, S. I.; Felder, F. D.; Poltorak, V.; Mewalal, R.; Blaby, I. K.; Robinson, S. L., Enzymatic carbon–fluorine bond cleavage by human gut microbes. *Proceedings of the National Academy of Sciences* **2025,** *122* (24), e2504122122.
11. Huang, S.; Jaffé, P. R., Defluorination of perfluorooctanoic acid (PFOA) and perfluorooctane sulfonate (PFOS) by Acidimicrobium sp. strain A6. *Environmental science & technology* **2019,** *53* (19), 11410-11419.
12. Yu, Y.; Zhang, K.; Li, Z.; Ren, C.; Chen, J.; Lin, Y.-H.; Liu, J.; Men, Y., Microbial cleavage of C–F bonds in two C6 per-and polyfluorinated compounds via reductive defluorination. *Environmental science & technology* **2020,** *54* (22), 14393-14402.
13. Oberortner, E.; Cheng, J.-F.; Hillson, N. J.; Deutsch, S., Streamlining the design-to-build transition with build-optimization software tools. *ACS synthetic biology* **2017,** *6* (3), 485-496.
14. Boeckmann, B.; Bairoch, A.; Apweiler, R.; Blatter, M.-C.; Estreicher, A.; Gasteiger, E.; Martin, M. J.; Michoud, K.; O'Donovan, C.; Phan, I., The SWISS-PROT protein knowledgebase and its supplement TrEMBL in 2003. *Nucleic acids research* **2003,** *31* (1), 365-370.
